# Efficacy and Safety of Ertugliflozin in Type 2 Diabetes: A Systematic Review and Meta-Analysis

**DOI:** 10.3389/fphar.2021.752440

**Published:** 2022-01-20

**Authors:** Li Liu, Fang-Hong Shi, Hua Xu, Yue Wu, Zhi-Chun Gu, Hou-Wen Lin

**Affiliations:** ^1^ Department of Pharmacy, Ren Ji Hospital, Shanghai Jiao Tong University School of Medicine, Shanghai, China; ^2^ Department of Endocrinology, Ren Ji Hospital, Shanghai Jiao Tong University School of Medicine, Shanghai, China; ^3^ Department of Pharmacy, Renmin Hospital, Wuhan University, Wuhan, China; ^4^ School of Medicine, Tongji University, Shanghai, China

**Keywords:** ertugliflozin, sodium-glucose cotransporter type-2 inhibitor, glycaemia, adverse drug event, meta-analysis

## Abstract

**Objective:** To evaluate the efficacy and safety of ertugliflozin in patients with type 2 diabetes.

**Methods:** MEDLINE, EMBASE, and Cochrane Library were searched (July 31, 2021) for phase II/III randomized clinical trials, which reported the efficacy and safety of ertugliflozin. Continuous variables were calculated as weighted mean difference (WMD) and associated 95% confidence intervals (CIs); dichotomous data were expressed as risk ratios (RRs) with 95% CIs.

**Results:** Nine randomized clinical trials including 5638 type 2 diabetes patients were included. For efficacy, ertugliflozin significantly reduced HbA1c (%) (WMD −0.452%; 95% CI −0.774 to −0.129), fasting plasma glucose (FPG) (WMD −0.870 mmol/L; 95% CI −1.418 to −0.322), body weight (WMD −1.774 kg; 95% CI −2.601 to −0.946), and blood pressure levels (systolic blood pressure: WMD −2.572 mmHg; 95% CI −3.573 to −1.571 and diastolic blood pressure: WMD −1.152 mmHg; 95% CI −2.002 to −0.303) compared with placebo and other hypoglycaemic agents. Compared with placebo, ertugliflozin was superior in reducing HbA1c (%) (WMD −0.641%) and FPG (WMD −1.249 mmol/L). And compared with active agents, ertugliflozin also could decrease HbA1c by 0.215% and FPG by 0.266 mmol/L. The interactive effect between different controls was significant (*P*_interaction_ of 0.039). For safety, similar to other sodium-glucose cotransporter type-2 inhibitors, ertugliflozin mainly increased the risk of genital mycotic infection (RR: 4.004; 95% CI 2.504–6.402). There was no significant difference in the incidence of any adverse events (AEs), AEs related to study drug, serious AEs, deaths, and discontinuations due to AEs. Results were consistent with the most primary outcomes in subgroups analysis and sensitivity analysis.

**Conclusion:** Ertugliflozin was relatively effective and tolerated in patients with type 2 diabetes compared with placebo or other hypoglycaemic agents, except for a high risk of genital mycotic infection.

**Systematic Review Registration:** (ClinicalTrials.gov), identifier (CRD42020206356).

## Introduction

Diabetes is a common chronic disease worldwide and is associated with adverse socio-economic outcomes (Bommer et al., 2017). The estimated population of adults with diabetes will rise to 642 million by 2040, of which 90% will be type 2 diabetes (Chatterjee et al., 2017). Metformin is recommended as the first-line therapy for patients with type 2 diabetes in the American Diabetes Association guidelines in 2021 (Tse et al., 2020). Sodium-glucose cotransporter type-2 (SGLT2) inhibitors, as a novel class of hypoglycaemic drugs, are recommended for their favourable effects on patients with type 2 diabetes, especially for the established risk of cardiovascular or renal complications (Chen et al., 2020).

Ertugliflozin is the fourth SGLT2 inhibitor approved by the US Food and Drug Administration in December 2017 for patients with type 2 diabetes (Markham, 2018). The absorption of ertugliflozin was rapid and complete, with time to the peak plasma concentration (*T*_max_) occurring at 1–2 h post-dose and nearly 100% oral bioavailability. The half-life (t_1/2_) of ertugliflozin ranged from 11–18 h, making it appropriate for once-daily administration (Fediuk et al., 2020). As a potent inhibitor of SGLT2, ertugliflozin reduces plasma glucose and glycated haemoglobin (HbA1c) levels by increasing urinary glucose excretion without inducing excessive insulin secretion in patients with type 2 diabetes (Derosa and Maffioli, 2018).

A recent network meta-analysis determined that ertugliflozin might be more efficacious in reducing HbA1c than dapagliflozin and empagliflozin (McNeill et al., 2019), with acceptable tolerability. However, the ability of ertugliflozin on glycaemia control, body weight, blood pressure, and the risk of drug-related adverse events had not been fully quantified. Previous meta-analyses on the efficacy or safety of ertugliflozin mainly focused on either the specific population or aspect (blood pressure, renal function, or safety) (Liu et al., 2019a; Liu et al., 2020b; Cherney et al., 2020; Patel et al., 2020), failing to comprehensively evaluate the efficacy and safety of ertugliflozin. Given above, in this study, we aimed to assess the effectiveness and safety of ertugliflozin for type 2 diabetes patients by integrating and quantifying all available evidence.

## Methods

This meta-analysis was conducted according to the Preferred Reporting Items for Systematic Review and Meta-Analysis statement (Shamseer et al., 2015) and a prior protocol at the International Prospective Register of Systematic Reviews (PROSPERO: CRD42020206356).

### Search Strategy

MEDLINE, EMBASE, and Cochrane Central Register of Controlled Trials were systematically searched to identify potentially eligible studies until July 31, 2021. We also searched ClinicalTrials.gov to identify unpublished studies. The search results were restricted to clinical trials and English publications. Cited references, reviews, and meta-analyses were checked to identify additional studies. Details of the study selection process are shown in Supplementary Table S1.

### Study Selection and Outcomes

Studies were considered if they met the following inclusion criteria: 1) Randomized controlled trials (RCTs); 2) investigation of adult patients with type 2 diabetes; 3) ertugliflozin; 4) reporting the efficacy and safety endpoints; and 5) duration of intervention of at least 12 weeks. Exclusion criteria for studies were as follows: conference abstracts, reviews, letters, editorials, case reports, observation studies, long-term extension studies, and post hoc analyses. The primary efficacy outcomes comprised glycaemic control [HbA1c, the proportion of participants achieving an HbA1c level < 7%, and fasting plasma glucose (FPG)]; weight loss (body weight); and blood pressure control [systolic blood pressure (SBP) and diastolic blood pressure (DBP)]. Adverse events (AEs) with different degrees and prespecified AEs of SGLT2 inhibitors were selected to assess the safety and tolerability of ertugliflozin. The primary safety outcomes were any AEs, AEs related to study drug, serious AEs, deaths, discontinuations due to AEs, and predetermined AEs of interest for ertugliflozin [genital mycotic infection (GMI), urinary tract infection (UTI), symptomatic hypoglycaemia, and hypovolaemia]. AEs included in the analysis were mainly coded according to the Medical Dictionary for Regulatory Activities, as defined in the individual study (Supplementary Table S2). Considering the resource costs and social benefits of type 2 diabetes, we performed a cost-effectiveness analysis of ertugliflozin based on information provided by the included clinical literature. Detailed methods, results, and discussion were presented in Supplementary Material.

### Data Extraction

Using designed electronic forms, two authors (LL and F-HS) extracted the data for each article, including the first author’s name, publication time, NCT number, randomisation, intervention characteristics (type, dose, and duration of interventions), patient characteristics (background treatment, the proportion of men, mean age, duration of type 2 diabetes), and reported outcomes. Any dispute was resolved by consensus or by consultation with the corresponding authors (YW and Z-CG).

### Quality Assessment

Two independent reviewers (LL and F-HS) performed the quality assessment using the Cochrane Collaboration’s tool (Higgins et al., 2011), and any disagreements were resolved by the corresponding authors (YW and Z-CG). Considering the risk of bias, we evaluated the following aspects: adequacy of random sequence generation, allocation concealment, blinding of participants and personnel, blinding of outcome assessment, completeness of outcome data, selective outcome reporting, and other biases that could induce confounding effects.

### Data Synthesis and Analysis

All statistical analyses were performed using Stata version 12.0 (Stata Corporation, College Station, TX, United States). Meta-analysis estimates of the studies were derived and presented as forest plots. We applied a random-effects model to evaluate the overall estimated effects. Continuous variables, including least-squares, mean change versus control group for HbA1c, FPG, body weight, SBP, and DBP, were calculated as weighted mean difference (WMD) and associated 95% confidence intervals (CIs). A WMD less than 0 signified that the results favoured the use of ertugliflozin compared with other therapies. For dichotomous data, including patients with HbA1c < 7% and the rate of any AEs, we used risk ratios (RRs) and their 95% CIs. An RR less than 1 indicated a lower trend in ertugliflozin treatment than that in control. Subgroup analyses were performed for overall efficacy and safety outcomes by dosage (ertugliflozin 5 mg, ertugliflozin 15 mg), follow-up (≤ 26 weeks, > 26 weeks), and control (placebo or active agents). The interaction analysis (*P* for interaction) was performed to assess the comparability of efficacy and safety indexes in different dosages, follow-ups, and controls (Gu et al., 2020). A significant *p* indicated the difference among subgroups. Further leave-one-out and fixed-effect model sensitivity analyses were applied to detect the robustness of the results. The *I* ^*2*^ test was used to measure the total variation between studies to test the heterogeneity among studies (significance for *I* ^*2*^ > 50%) (Higgins and Thompson, 2002). Statistical significance was set at *p* < 0.05.

## Results

### Study Selection and Characteristics

**FIGURE 1:**
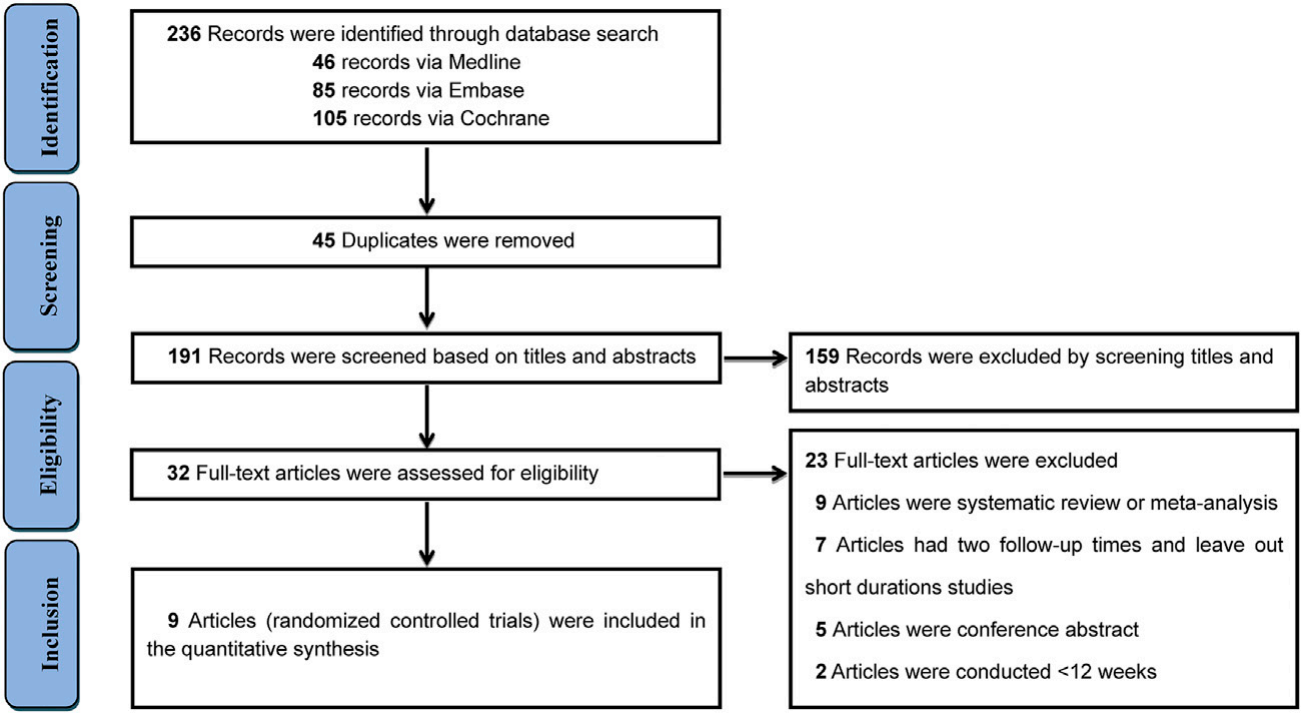
PRISMA diagram of the selection of eligible randomized controlled trials.

The initial search identified 236 records meeting the inclusion criteria, and nine studies (RCT trials) involving 5638 participants were finally included in the quantitative synthesis (Amin et al., 2015; Aronson et al., 2018; Dagogo-Jack et al., 2018; Grunberger et al., 2018; Miller et al., 2018; Pratley et al., 2018; Gallo et al., 2019; Hollander et al., 2019; Ji et al., 2019). The screening process is illustrated in Figure 1. The publication years of included trials were from 2015 to 2019. Background treatments included diet and exercise, metformin, and other antihyperglycaemic agents. Of the included trials, five trials were controlled with placebo (Amin et al., 2015; Dagogo-Jack et al., 2018; Grunberger et al., 2018; Miller et al., 2018; Ji et al., 2019), two trials were compared with glimepiride (Gallo et al., 2019; Hollander et al., 2019), and the remaining two were controlled with metformin, sitagliptin, respectively (Aronson et al., 2018; Pratley et al., 2018). Seven trials mainly focused on Caucasians (ranged from 64.8 to 90.4%), one trial focused on Asians (406 Chinese patients), and one trial did not report races. The duration of the trial follow-up ranged from 12 to 104 weeks. The mean age of participants was 57.6 years, and the mean duration of diabetes was 4.6–14.7 years. The mean HbA1c % ranged from 7.8 to 9.0%. Detailed demographic characteristics are presented in Table 1.

**TABLE 1:** Characteristics of included RCTs of ertugliflozin.

Study	Amin et al. (2015)		Aronson et al. (2018)		Dagogo-Jack et al. (2018)		Gallo et al. (2019)		Grunberger et al. (2018)		Hollander et al. (2019)		Ji et al. (2019)		Miller et al. (2018)		Pratley et al. (2018)	
NCT number	01059825		01958671		02036515		02033889		01986855		01999218		02630706		02226003		02099110	
Follow-up (weeks)	12		52		52		104		52		104		26		26		52	
Background	MET		DE		MET + SIT		DE + MET		DE ± AHA		MET		MET		DE		MET	
Control	PLA/SIT	ERT	PLA/MET	ERT	PLA	ERT	PLA/GLI	ERT	PLA	ERT	GLI	ERT	PLA	ERT	PLA	ERT + SIT	SIT	ERT
Participants	109	219	153	308	153	309	209	412	154	313	437	888	167	339	97	194	247	985
Male sex (%)	64.2	65.3	53.6	58.1	65.4	52.7	46.9	46.2	46.8	50.8	51.3	47.1	52.7	56.9	58.8	56.7	62.3	51.8
Mean age (year)	53.6	54.8	56.1	56.5	58.3	59.4	56.5	56.7	67.5	67.1	57.8	58.4	56.9	56.2	54.3	56.3	54.8	55.2
Mean duration of diabetes (year)	6.3	6.3	4.6	5.2	9.4	9.6	8.0	8.0	13.1	14.7	7.5	7.4	6.4	7.2	6.8	6.1	6.2	7.1
HbA1c (%)	8.2	8.1	8.1	8.3	8.0	8.1	8.2	8.1	8.1	8.2	7.8	7.8	8.1	8.1	9.0	8.9	8.5	8.6
HbA1c (mmol/mol)	NK	NK	65.2	66.7	64.3	64.3	NK	NK	NK	NK	61.3	61.9	NK	NK	74.3	74.1	69.4	70.1
Body weight (kg)	NK	NK	94.2	92.3	86.4	87.1	84.5	85.1	90.4	87.6	86.8	86.8	70.1	70.5	95.0	91.0	89.8	88.4
BMI (kg/m^2^)	30.5	30.4	33.3	32.9	30.3	31.1	30.7	30.9	33.2	32.2	31.2	31.5	26.1	25.9	32.7	32.0	31.7	31.9
eGFR (mL/min/1.73 m^2^)	NK	NK	86.2	88.4	89.9	87.0	91.6	89.9	46.0	46.8	86.6	87.5	99.9	99.0	92.6	89.8	92.6	92.3
SBP (mmHg)	126.6	126.3	129.8	130.1	130.2	131.9	129.3	130.4	NK	NK	129.9	130.5	NK	NK	127.4	130.0	128.3	129.5
DBP (mmHg)	79.2	78.6	78.1	78.5	78.5	78.6	77.5	78.3	NK	NK	NK	NK	NK	NK	77.8	77.6	NK	NK
FPG (mmol/L)	9.2	9.1	10.0	10.0	9.4	9.4	9.4	9.3	8.7	8.8	8.8	9.0	9.2	9.4	11.5	10.7	9.8	10.1
Race (%)																		
Asian	NK	NK	9.8	7.8	21.6	19.8	14.8	16.7	5.8	11.5	16.7	18.7	100.0	100.0	0	0	11.7	10.4
Black or African American	NK	NK	5.9	6.5	2.0	1.9	9.1	10.9	2.6	4.8	5.7	4.0	0	0	4.1	4.6	4.5	3.5
White	NK	NK	82.4	84.4	70.6	74.1	68.9	64.8	87.0	78.6	72.8	73.0	0	0	92.8	89.2	78.1	80.8
Others*	NK	NK	2.0	1.3	5.9	4.2	7.2	7.5	4.5	5.1	4.8	4.2	0	0	3.1	6.2	5.6	5.3

MET, metformin; DE, diet and exercise; SIT, sitagliptin; AHA, antihyperglycaemic agent; PLA, placebo; ERT, ertugliflozin; GLI, glimepiride; HbA1c, glycated haemoglobin; FPG, fasting plasma glucose; BMI, body mass index; eGFR, estimated glomerular filtration rate; SBP, systolic blood pressure; DBP, diastolic blood pressure; NK, not known. * Others includes American Indian or Alaska Native, multiple, and Native Hawaiian or other Pacific Islander.

### Overall Efficacy Outcomes

**FIGURE 2:**
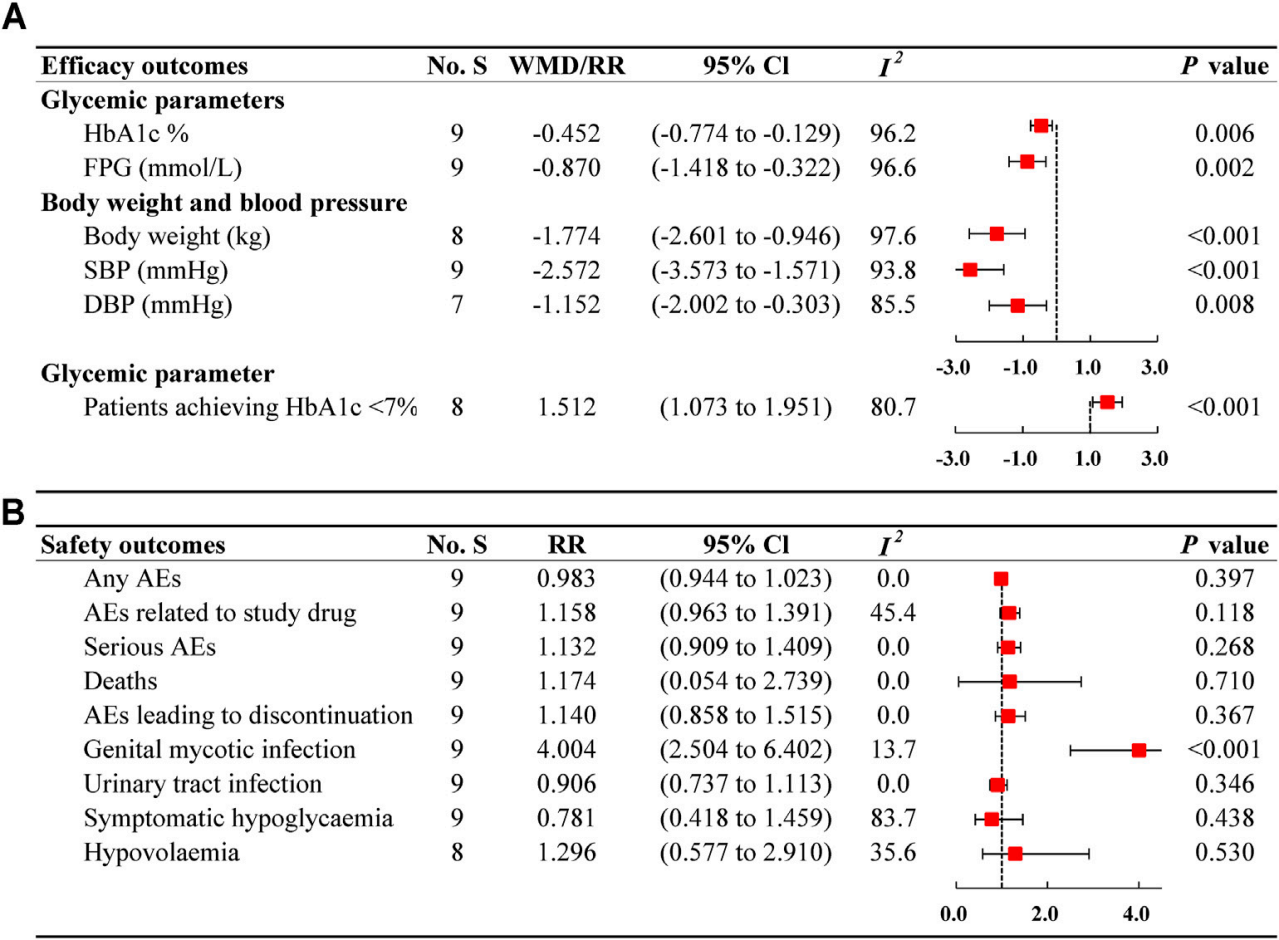
Forest plots of ertugliflozin on efficacy **(A)** and safety **(B)** outcomes. No. S, numbers of studies; WMD, weighted mean difference; CI, confidence interval; I2, heterogeneity; HbA1c, glycated haemoglobin; FPG, fasting plasma glucose; SBP, systolic blood pressure; DBP, diastolic blood pressure; RR, risk ratio; AEs, adverse events.

We evaluated the effects of ertugliflozin on glycaemic variables, body weight, and blood pressure, as shown in Figure 2. Meta-analysis results revealed that ertugliflozin significantly decreased HbA1c levels (%) (WMD −0.452%; 95% CI −0.774 to −0.129; *I* ^*2*^ = 96.2%), HbA1c (mmol/mol) (WMD −7.722 mmol/mol; 95% CI −14.691 to −0.753; *I* ^*2*^ = 98.3%), FPG (WMD −0.870 mmol/L; 95% CI −1.418 to −0.322; *I* ^*2*^ = 96.6%), consequently increasing the rate of patients achieving target HbA1c (< 7%) (RR: 1.152; 95% CI: 1.073–1.951; *I* ^*2*^ = 80.7%), compared with other hypoglycaemic agents or placebo. For body weight and blood pressure, the reduction in body weight from baseline was more considerable in ertugliflozin than non-ertugliflozin (WMD −1.774 kg; 95% CI −2.601 to −0.946; *I* ^*2*^ = 97.6%). A greater reduction in blood pressure levels was also observed in patients receiving ertugliflozin compared with other treatments (SBP: WMD −2.572 mmHg; 95% CI −3.573 to −1.571; *I* ^*2*^ = 93.8% and DBP: WMD −1.152 mmHg; 95% CI −2.002 to −0.303; *I* ^*2*^ = 85.5%).

### Subgroup Analysis and Sensitivity Analysis of Efficacy Outcomes

Table 2 shows the results of the subgroup analysis on the regulation of glycaemia, blood pressure, and body weight. Regarding glycaemic control, the reduction in HbA1c (%) and FPG based on different dosages were in line with the primary outcomes. For follow-up duration, patients administered with ertugliflozin decreased HbA1c mainly in short follow-up studies, rather than in long ones (≤ 26 weeks: WMD −0.788%; 95% CI −1.169 to −0.407; *I* ^*2*^ = 88.6%; >26 weeks: WMD −0.287%; 95% CI −0.646 to 0.072; *I* ^*2*^ = 95.6%; *P*_interaction_ of follow-ups = 0.061). For different controls, ertugliflozin presented a notable decrease in HbA1c compared with placebo (WMD −0.641%; 95% CI −0.984 to −0.298; *I* ^*2*^ = 93.0%) and active agents (WMD −0.215%; 95% CI −0.629 to −0.199; *I* ^*2*^ = 94.9%), but the interactive effect was significant (*P*_interaction_ of 0.039). Additionally, ertugliflozin had a significant reduction in FPG levels for less than 26 weeks compared with more than 26 weeks, with a *P*_interaction_ of 0.023. Similar results were also found in different controls (*P*_interaction_ of 0.006). As for reaching the glycaemia target, subgroup analyses showed that when used less than 26 weeks or compared with placebo, ertugliflozin contributed to a higher proportion of patients attaining HbA1c (< 7%), with the risk ratio of 4.045 (95% CI: 2.098–5.992; *I* ^*2*^ = 41.0%), 3.834 (95% CI: 2.677–4.991; *I* ^*2*^ = 11.6%), respectively. The interactive effects were of significant difference when follow-ups and controls were considered (*P*_interaction_ < 0.05). For different dosages, the results of subgroup analyses (5 mg RR: 1.196; 95% CI: 0.843–1.549; *I* ^*2*^ = 64.9%; 15 mg RR: 1.326; 95% CI: 0.908–1.744; *I* ^*2*^ = 69.2%) were not consistent with the primary results (RR 1.152; 95% CI: −1.073 to 1.951; *I* ^*2*^ = 80.7%). Further, the results of leave-one-out sensitivity analysis were basically similar to the primary outcomes. Concerning the proportion of patients achieving HbA1c < 7%, two trials were found to influence the primary outcomes of individual trials (Dagogo-Jack et al., 2018; Ji et al., 2019) (Supplementary Table S3).

**TABLE 2:** Subgroup analysis of main efficacy outcomes of ertugliflozin vs. control.

Subgroup		No. S	WMD	95% CI	*I* ^2^	PI
Subgroup No. S RR95% I2 PI Different dosages						
HbA1c%	5 mg	8	−0.434	−0.790 to −0.078	94.4%	0.886
	15 mg	8	−0.469	−0.789 to −0.150	93.2%	
FPG	5 mg	8	−0.825	−1.421 to −0.229	95.2%	0.738
	15 mg	8	−0.962	−1.498 to −0.426	93.7%	
Body weight	5 mg	8	−1.750	−2.652 to −0.848	96.0%	0.931
	15 mg	8	−1.807	−2.719 to −0.896	96.0%	
SBP	5 mg	8	−1.956	−2.828 to −1.083	88.0%	0.516
	15 mg	8	−1.556	−2.388 to −0.723	87.7%	
DBP	5 mg	6	−0.877	−1.695 to −0.059	61.6%	0.579
	15 mg	6	−0.586	−1.209 to 0.037	38.3%	
Different follow-ups						
HbA1c%	≤26 weeks	3	−0.788	−1.169 to −0.407	88.6%	0.061
	>26 weeks	6	−0.287	−0.646 to 0.072	95.6%	
FPG	≤26 weeks	3	−1.581	−2.352 to −0.809	89.2%	0.023
	>26 weeks	6	−0.522	−1.012 to −0.032	94.3%	
Body weight	≤26 weeks	2	−1.929	−2.243 to −1.615	0%	0.677
	>26 weeks	6	−1.705	−2.710 to −0.699	98.0%	
SBP	≤26 weeks	3	−4.154	−5.627 to −2.681	50.6%	0.006
	>26 weeks	6	−1.676	−2.628 to −0.724	92.5%	
DBP	≤26 weeks	3	−2.042	−2.812 to −1.271	18.7%	0.006
	>26 weeks	4	−0.566	−1.294 to 0.161	69.7%	
Different controls						
HbA1c%	placebo	5	−0.641	−0.984 to −0.298	93.0%	0.039
	active	4	−0.215	−0.629 to −0.199	94.9%	
FPG	placebo	5	−1.249	−1.895 to −0.602	92.4%	0.006
	active	4	−0.266	−0.518 to −0.013	73.1%	
Body weight	placebo	4	−1.954	−2.221 to −1.686	0%	0.565
	active	4	−1.578	−2.831 to −0.324	98.7%	
SBP	placebo	5	−4.177	−5.160 to −3.195	19.3%	<0.001
	active	4	−0.974	−1.829 to −0.118	92.3%	
DBP	placebo	4	−1.756	−2.546 to −0.967	38.8%	0.037
	active	3	−0.504	−1.374 to 0.365	76.8%	
Subgroup No.S RR95% I2 PI Different dosages						
Patients achieving HbA1c <7%	5 mg	7	1.196	0.843–1.549	64.9%	0.641
	15 mg	7	1.326	0.908–1.744	69.2%	
Different follow-ups						
Patients achieving HbA1c <7%	≤26 weeks	3	4.045	2.098–5.992	41.0%	0.005
	>26 weeks	5	1.211	0.877–1.545	76.6%	
Different controls						
Patients achieving HbA1c <7%	placebo	4	3.834	2.677–4.991	11.6%	<0.001
	active	4	1.093	0.864–1.322	58.4%	

No. S, numbers of studies; WMD, weighted mean difference; PI, *P* for interaction; RR, risk ratio; HbA1c, glycated haemoglobin; FPG, fasting plasma glucose; SBP, systolic blood pressure; DBP, diastolic blood pressure.

For body weight and SBP, the results of subgroup analyses across dosage, follow-up, or control were all in line with the primary outcomes. Regarding DBP, the overall subgroup analyses were not completely consistent with the primary analyses. For different dosages considered, only the use of 5 mg ertugliflozin was found to significantly decrease DBP levels (WMD −0.877 mmHg; 95% CI −1.695 to −0.059; *I* ^*2*^ = 61.6%), although the use of 15 mg ertugliflozin was associated with a decreasing trend (WMD −0.586 mmHg; 95% CI −1.209 to 0.037; *I* ^*2*^ = 38.3%), with a *P*_interaction_ of 0.579. For follow-up duration, a meaningful reduction of DBP was observed when ertugliflozin was used less than 26 weeks (WMD −2.042 mmHg; 95% CI −2.812 to 1.271; *I* ^*2*^ = 69.7%). By contrast, ertugliflozin treatment more than 26 weeks failed to decrease DBP levels (WMD −0.566 mmHg; 95% CI −1.294 to 0.161; *I* ^*2*^ = 69.7%) (*P*_interaction_ of 0.006). Similar results were observed in the subgroup analysis of different controls, and a *P*_interaction_ between placebo and active controls was 0.037. Additionally, further sensitivity analyses on body weight and blood pressure indicated that the outcomes remained invariant whatever either study was excluded. The results of the fixed-effect model remained consistent with that of the random-effect model (Supplementary Table S4).

### Safety Outcomes

The analysis assessed the tolerability of type 2 diabetes patients, as presented in Figure 2. The incidence of any AEs was 63.58% (2517/3959) in ertugliflozin group and 65.73% (1097/1669) in non-ertugliflozin group (Supplementary Table S5), indicating a similar risk between the two therapies (RR: 0.983; 95% CI 0.944–1.023; *I* ^*2*^ = 0%). The results were consistent with those of all subgroups, as shown in Table 3. Also, no significant differences between ertugliflozin group and control group were observed in terms of serious AEs (RR: 1.132; 95% CI 0.909–1.409; *I* ^*2*^ = 0%), death (RR: 1.174; 95% CI 0.054–2.739; *I* ^*2*^ = 0%), and AEs leading to discontinuation (RR: 1.140; 95% CI 0.858–1.515; *I* ^*2*^ = 0%). There were no notable differences between groups in the incidence of AEs related to study drug (1.158; 95% CI 0.963–1.391; *I* ^*2*^ = 45.4%), although the incidence was higher when using ertugliflozin (19.22%) compared with other therapies (17.14%).

**TABLE 3:** Subgroup analysis of main safety outcomes of ertugliflozin vs. control.

Subgroup		No. S	RR	95% CI	*I* ^*2*^	PI
Different dosages						
Any AEs	5 mg	8	0.994	0.950–1.041	0%	0.586
	15 mg	8	0.976	0.931–1.023	0%	
AEs related to study drug	5 mg	8	1.148	0.914–1.442	53.6%	0.921
	15 mg	8	1.168	0.913–1.495	59.9%	
Serious AEs	5 mg	8	1.219	0.952–1.559	0%	0.489
	15 mg	8	1.069	0.812–1.407	8.2%	
Deaths	5 mg	4	1.466	0.407–5.278	29.1%	0.875
	15 mg	4	1.240	0.469–3.283	0%	
AEs leading to discontinuation	5 mg	8	1.155	0.836–1.596	0%	0.980
	15 mg	8	1.148	0.826–1.597	0%	
Genital mycotic infection	5 mg	8	4.094	2.588–6.476	1.8%	0.882
	15 mg	8	4.338	2.480–7.588	26.2%	
Urinary tract infection	5 mg	8	0.845	0.601–1.189	39.5%	0.490
	15 mg	8	0.982	0.760–1.269	10.2%	
Symptomatic hypoglycaemia	5 mg	8	0.708	0.336–1.492	83.8%	0.912
	15 mg	8	0.750	0.416–1.354	75.1%	
Hypovolaemia	5 mg	8	1.466	0.714–3.010	19.1%	0.934
	15 mg	8	1.403	0.739–2.663	1.5%	
Different follow-ups						
Any AEs	≤26 weeks	3	0.966	0.854–1.093	0%	0.768
	>26 weeks	6	0.985	0.944–1.027	0%	
AEs related to study drug	≤26 weeks	3	1.229	0.869–1.738	0%	0.761
	>26 weeks	6	1.149	0.906–1.458	64.3%	
Serious AEs	≤26 weeks	3	1.512	0.283–8.078	65.4%	0.845
	>26 weeks	6	1.122	0.895–1.406	0%	
Deaths	>26 weeks	5	1.174	0.504–2.739	0%	—
AEs leading to discontinuation	≤26 weeks	3	0.732	0.280–1.910	0%	0.314
	>26 weeks	6	1.190	0.883–1.602	0%	
Genital mycotic infection	≤26 weeks	3	1.977	0.756–5.165	0%	0.112
	>26 weeks	6	4.962	2.826–8.712	23.4%	
Urinary tract infection	≤26 weeks	3	0.820	0.398–1.687	0%	0.810
	>26 weeks	6	0.904	0.698–1.171	27.8%	
Symptomatic hypoglycaemia	≤26 weeks	3	3.896	1.058–14.337	0%	0.329
	>26 weeks	6	0.580	0.305–1.102	87.2%	
Hypovolaemia	≤26 weeks	2	1.170	0.211–6.474	0%	0.898
	>26 weeks	6	1.397	0.560–3.484	51.9%	
Different controls						
Any AEs	placebo	5	0.966	0.903–1.033	0%	0.533
	active	4	0.992	0.944–1.043	0%	
AEs related to study drug	placebo	5	1.251	0.984–1.591	0%	0.571
	active	4	1.115	0.812–1.531	76.6%	
Serious AEs	placebo	5	1.159	0.682–1.971	30.8%	0.930
	active	4	1.127	0.853–1.489	0%	
Deaths	active	4	1.192	0.400–3.559	0%	—
AEs leading to discontinuation	placebo	5	0.995	0.594–1.667	0%	0.549
	active	4	1.205	0.850–1.707	2.8%	
Genital mycotic infection	placebo	5	2.725	1.287–5.768	0%	0.272
	active	4	5.142	2.640–10.016	35.6%	
Urinary tract infection	placebo	5	0.710	0.490–1.030	0%	0.156
	active	4	1.004	0.745–1.353	27.9%	
Symptomatic hypoglycaemia	placebo	5	1.152	0.668–1.988	19.9%	0.075
	active	4	0.464	0.224–0.963	82.7%	
Hypovolaemia	placebo	4	1.237	0.299–5.119	33.4%	0.917
	active	4	1.392	0.519–3.737	53.8%	

No. S, numbers of studies; RR, risk ratio; PI, *P* for interaction; AEs, adverse events.

Regarding GMI, ertugliflozin use increased the 3-fold risk compared with other medications (RR: 4.004; 95% CI 2.504–6.402; *I* ^*2*^ = 13.7%), with a higher incidence in ertugliflozin group (6.59%) versus in control group (1.44%). However, when the follow-up was less than 26 weeks, no correlation of higher GMI risk was observed with ertugliflozin treatment (RR: 1.977; 95% CI 0.756–5.165; *I* ^*2*^ = 0%) (Table 3). AEs related to symptomatic hypoglycaemia were more common in non-ertugliflozin group (10.84%) than in ertugliflozin group (5.35%). However, there was no evidence that ertugliflozin use had a lower risk of symptomatic hypoglycaemia than other therapies (RR: 0.781; 95% CI 0.418–1.459; *I* ^*2*^ = 83.7%). In the subgroup analysis by follow-up, a higher risk of symptomatic hypoglycaemia in ertugliflozin group was found in less than 26 weeks compared with in non-ertugliflozin group (RR: 3.896; 95% CI 1.058–14.337; *I* ^*2*^ = 0%). We failed to find a higher risk of UTI in ertugliflozin treatment group compared with the control (RR: 0.906; 95% CI 0.737–1.113; *I* ^*2*^ = 0%), with a lower incidence (6.92% in ertugliflozin group, 7.67% in control group, respectively). Also, there was no meaningful difference between the two groups in hypovolemia (RR: 1.296; 95% CI 0.577–2.910; *I* ^*2*^ = 35.6%), with a comparable incidence (1.66% in ertugliflozin group, 1.18% in control group). Further, the results were similar in fixed-effect model sensitive analysis (Supplementary Table S6).

### Risk of Bias

Seven trials used an interactive voice response system/integrated web response system when allocating concealment; two trials that did not report a similar system were evaluated as unclear. One trial that did not report the personnel assessing the outcomes was unclear. According to the bias tool item, nine trials were assessed to have a low risk of bias (Supplementary Table S7).

## Discussion

### Major Findings

This study involved 5638 patients with type 2 diabetes from nine randomized clinical trials to evaluate the efficacy and safety of ertugliflozin comprehensively. Overall, the results revealed that ertugliflozin performed well in glycaemic control, blood pressure and weight management compared with other therapies (placebo, metformin, glimepiride, and sitagliptin). Ertugliflozin presented a better profile of glycaemic control and blood pressure regulation controlled with placebo rather than other active agents. For safety, ertugliflozin provided favourable tolerability in patients with type 2 diabetes but increased the risk of GMI, urging for personal hygiene education and close supervision in clinical treatment. The results of key subgroups were mainly consistent with the primary outcomes.

### Glycaemia Control

Ertugliflozin is a highly selective inhibitor with remarkable selectivity for SGLT2 over SGLT1 (> 2000-fold) (Cinti et al., 2017). Its oral bioavailability was nearly 100%, which was higher than that of dapagliflozin (78%) and canagliflozin (65%) (Kasichayanula et al., 2014; Devineni and Polidori, 2015; Fediuk et al., 2020). Therefore, ertugliflozin may have a preferable hypoglycaemic effect. We demonstrated that ertugliflozin lowered 0.45% HbA1c and raised 15.2% of the proportion of patients achieving target HbA1c (< 7%) than placebo and other glucose-lowering agents. Ertugliflozin resulted in a more significant reduction of HbA1c (%) in placebo-controlled groups than active agents-controlled ones, with a *P*_interaction_ of controls 0.039. It was reasonable and confirmed the credibility of our analysis. Meanwhile, subgroup analysis showed that 5 and 15 mg ertugliflozin reduced HbA1c by 0.43 and 0.47%, respectively. Generally, our results remained consistent with previous meta-analyses of ertugliflozin, in which ertugliflozin reduced HbA1c by 0.5–1.0% (Liu et al., 2020b; Cherney et al., 2020; Pratley et al., 2020). Analyses of three trials within 26 weeks showed ertugliflozin yielded the target HbA1c (< 7%) with a high RR of 4.045 (Amin et al., 2015; Miller et al., 2018; Ji et al., 2019). The results noted that ertugliflozin might exhibit better glycaemic control in the first few months. The mechanism is unclear, and the possible reason is that SGLT2 inhibitors seem to occur an early and rapid body water and fat loss up to approximately 8 weeks, then become a slower rate of sustained fat loss (Lee et al., 2018). Furthermore, a network meta-analysis performed by McNeill (McNeill et al., 2019) found that ertugliflozin was more effective in decreasing HbA1c than dapagliflozin and empagliflozin in patients with type 2 diabetes. When used alone, ertugliflozin 15 mg was more effective in reducing HbA1c than 10 mg dapagliflozin (0.36%) or 25 mg empagliflozin (0.31%). When combined with metformin, ertugliflozin was more effective than dapagliflozin (ertugliflozin 5 mg versus dapagliflozin 5 mg 0.22%; ertugliflozin 15 mg versus dapagliflozin 10 mg 0.26%). Furthermore, 15 mg ertugliflozin lowered HbA1c by 0.23% more than 25 mg empagliflozin. A model-based meta-analysis consolidated the results, which performed an indirect comparison of SGLT2 inhibitor efficacy, indicating that ertugliflozin was of comparable efficacy in magnitude to other SGLT2 inhibitors (Deryl Fediuk et al., 2019). Although there was no head-to-head study comparing the hypoglycaemic effect across all SGLT2 inhibitors, these results indicated that ertugliflozin had a relatively higher hypoglycaemic effect.

### Weight Loss and Blood Pressure Control

Obesity and hypertension are associated with cardiovascular morbidity and contribute to death in patients with type 2 diabetes (Low Wang et al., 2016). Improvement of these cardiovascular risk factors lowers the excess risk of death in adults with type 2 diabetes (Rawshani et al., 2018). SGLT2 inhibitors have a profile of weight loss and blood pressure control by a multifactorial mechanism (Chilton et al., 2015; Pfeifer et al., 2017). Weight loss might be directly related to glucose excretion in the kidneys by SGLT2 inhibitors, resulting in noticeable calorie loss (Pereira and Eriksson, 2019). The blood pressure drop caused by SGLT2 inhibitors is commonly explained by natriuresis and diuretic effects (Baker et al., 2017; Mazidi et al., 2017). SGLT2 inhibitors, by promoting urinary output, lead to increased urinary sodium excretion and some plasma volume contraction, resulting in blood pressure reduction (Mazidi et al., 2017).

Similarly, ertugliflozin might also induce weight loss and depressurisation as a class effect of SGLT2 inhibitors. Our analysis showed that ertugliflozin reduced body weight by 1.77 kg compared with other therapies. Moreover, ertugliflozin reduced blood pressure by 2.57 mmHg on SBP and 1.15 mmHg on DBP from baseline compared with control groups. In prior meta-analyses, ertugliflozin was also observed to reduce body weight by 0.9–3.5 kg (Liu et al., 2019b; Liu et al., 2020b; Heymsfield et al., 2020) and blood pressure (mainly SBP) by 1.8–7.2 mmHg (Liu et al., 2019a; Liu et al., 2020a; Pratley et al., 2020). These results are consistent with our findings, confirming the reliability of our results.

### Safety Profile

In this meta-analysis, ertugliflozin generally exhibited good tolerability in patients with type 2 diabetes, with an overall safety profile in monotherapy and combination therapy. There was no significant difference in the incidence of any AEs, AEs related to study drug, and serious AEs, and deaths and discontinuations due to AEs. Of the respecified AEs of interest for ertugliflozin, the incidence of UTI, symptomatic hypoglycaemia, and hypovolaemia failed to detect any significant difference. Subgroup analyses on symptomatic hypoglycaemia showed that ertugliflozin use within 26 weeks presented a greater relative risk with a wide 95% CI. The possible explanation was the favourable glycaemic control in the first few months. Our previous study, which included 78 relevant publications, found that SGLT2 inhibitors increased the risk of GMI (RR: 3.71) (Shi et al., 2019), of which six articles reported the risk of GMI (RR: 4.69) related to ertugliflozin. This issue was considered a class effect of SGLT2 inhibitors. The current study also confirmed that ertugliflozin was associated with a higher GMI risk (RR: 4.004). A greater risk of GMI was observed in more than 26 weeks (RR: 4.962) compared with less than 26 weeks (RR: 1.977). Results of subgroups by follow-up suggested that GMI risk of ertugliflozin might be related to a more prolonged therapeutic course, although the interactive effect was not found (*P*_interaction_ of 0.112). Generally, patients with type 2 diabetes are at an increased risk of infection, especially those with obesity or increased atherosclerotic plaque development (Peleg et al., 2007). Euglycaemia, BMI control, and decreased arteriosclerotic cardiovascular disease risk should be standard practices to reduce the risk of GMI. Thus, more attention should be paid before using ertugliflozin and close monitoring indicators clinically. Additionally, personal hygiene education is recommended for patients initiating SGLT2 inhibitors (Williams and Ahmed, 2019). If GMI occurred unavoidably with a treatment course, discontinuation of SGLT2 inhibitors was unnecessary for the usually mild infection, which could be resolved with oral antifungal or antifungal cream (Engelhardt et al., 2021).

### Clinical Implications

This meta-analysis quantified the efficacy and tolerability of ertugliflozin on glycaemia regulation, body weight control, blood pressure reduction, and incidence of AEs for type 2 diabetes patients. Thus, it seemed that ertugliflozin might be a favourable alternative to other SGLT2 inhibitors for type 2 diabetes inadequate responders.

### Strengths and Limitations

Our study comprehensively assessed ertugliflozin based on medication tolerability and clinical efficacy (with a relatively larger sample size), contributing to more effective clinical decision-making. The results of subgroups analysis and sensitivity analysis showed a basically consistent trend. Additionally, economic factors also matter when making clinical decisions for local medical providers. We performed a simple cost-effectiveness analysis of ertugliflozin to evaluate whether ertugliflozin had a favourable economic advantage (Supplementary Material). However, our study has some limitations. First, several outcomes showed moderate to high heterogeneity, and the baseline characteristics of included trials varied (different dosages, controls, or duration of follow-up), despite subgroup analyses and sensitivity analyses being conducted. Second, owing to the limited duration of follow-up, which ranged from 12 to 52 weeks mainly, a certain bias might exist undeniably, and the safety outcomes of ertugliflozin need a further long-term observation. Finally, a comparison between ertugliflozin and other SGLT2 inhibitors was not performed. Therefore, more comprehensive studies across all SGLT2 inhibitors would be meaningful and worth investigating.

## Conclusion

Our meta-analysis illustrated that ertugliflozin performed well in glycaemic control, weight loss, and blood pressure reduction. Ertugliflozin was relatively effective and tolerated in patients with type 2 diabetes, except for a high GMI risk. Given that, ertugliflozin may be a good alternative to other SGLT2 inhibitors. Further studies with head-to-head comparisons among SGLT2 inhibitors are needed to explore whether ertugliflozin would present a better profile of glycaemic control and safety outcomes.

## Data Availability

The original contributions presented in the study are included in the article/Supplementary Material, further inquiries can be directed to the corresponding authors.
