# Supplementary material for: Efficacy and Safety of Ertugliflozin in Type 2 Diabetes: A Systematic Review and Meta-Analysis
Source: Front Pharmacol. 2022 Jan 20;12:752440. doi: 10.3389/fphar.2021.752440 (PMC8811446; doi:10.3389/fphar.2021.752440)
Supplement: Supplementary file 1 [file DataSheet1.docx]

**Efficacy and safety of ertugliflozin in type 2 diabetes: A systematic review and meta-analysis**

[Cost-effectiveness analysis 1](#_Toc91686071)

[Supplementary tables 5](#_Toc91686072)

[Table S1. Search strategy used on 31 July 2021 5](#_Toc91686073)

[Table S2. Definition of adverse events (AEs) included in the analysis 6](#_Toc91686074)

[Table S3. Leave-one-out sensitivity analysis for main efficacy outcomes of ertugliflozin 8](#_Toc91686075)

[Table S4. Subgroup analysis of main efficacy outcomes of ertugliflozin versus control using a fixed-effect model 10](#_Toc91686076)

[Table S5. Summarized adverse events of interest in included studies 11](#_Toc91686077)

[Table S6. Subgroup analysis of main safety outcomes of ertugliflozin versus control using a fixed-effect model 12](#_Toc91686078)

[Table S7. Quality assessment results of included randomized controlled trials 13](#_Toc91686079)

[Table S8. PRISMA checklists 14](#_Toc91686080)

[Table S9. The costs data of medicines per day 17](#_Toc91686081)

[Table S10. Results of cost-effectiveness and sensitivity analyses (the percentage of qualified HbA1c as effective index) 18](#_Toc91686082)

[Table S11. Results of cost-effectiveness and sensitivity analyses (the decreased value of HbA1c % as effective index) 19](#_Toc91686083)

[Supplementary Figures 20](#_Toc91686084)

[Figure S1. Forest plots of ertugliflozin on efficacy outcomes (A: HbA1c%, B: FPG, and C: body weight) 20](#_Toc91686085)

[Figure S2. Forest plots of ertugliflozin on efficacy outcomes (A: SBP, B: DBP, and C: proportion of patients achieving HbA1c <7%) 21](#_Toc91686086)

[Figure S3. Forest plots of ertugliflozin on safety outcomes (A: Any AEs, B: AEs related to study drug, and C: serious AEs) 22](#_Toc91686087)

[Figure S4. Forest plots of ertugliflozin on safety outcomes (A: deaths, B: AEs leading to discontinuation, and C: GMI) 23](#_Toc91686088)

[Figure S5. Forest plots of ertugliflozin on safety outcomes (A: UTI, B: symptomatic hypoglycaemia, and C: hypovolaemia) 24](#_Toc91686089)

[Figure S6. Forest plots of ertugliflozin on efficacy and safety outcomes using a fixed-effect model 25](#_Toc91686090)

**Cost-effectiveness analysis**

**1. Methods**

The cost-effectiveness analysis was performed by calculating the incremental cost-effectiveness ratio (ICER). ICER presented an increase per unit of effect on study therapy. The treatment strategies would be assessed based on information provided by the included clinical literature. Effectiveness was measured in terms of the percentage of patients with HbA1c levels < 7% and the decreased value of HbA1c % from baseline (obtained from individual included trials). The analysis on the decreased value of HbA1c % is an alternative scenario analysis. Only direct medical costs were incorporated from the perspective of healthcare providers. The median drug costs were derived from the YAOZH database in China (<https://db.yaozh.com>), as shown in **Supplementary Table S7**. The medicine costs were calculated as follows: costs = average medicine costs daily × days. Due to the lack of a willingness-to-pay (WTP) threshold to determine cost-effectiveness in China, we used three times of China’s per capita gross domestic product (GDP) as the WTP threshold (WHO, 2001). The per capita GDP of China is 10503.52 USD in 2020 (Collinge et al., 2018; Kremer et al., 2018). In other words, the cost-effectiveness WTP threshold was $31510.56. Additionally, we conducted a one-way sensitivity analysis. We allowed the effects to vary between the upper and lower limits of 95% CI and the drug cost to vary by a range of ± 10 and ± 20% (Sathish et al., 2020).

**2. Results**

We used the proportion of participants achieving an HbA1c level < 7% (**Supplementary Table S8**) and the decreased value of HbA1c % (**Supplementary Table S9**) as the effective indexes in cost-effectiveness analysis. Similar results were observed in two effective indexes. The former results were shown here. Five scenarios were considered in the economic evaluation of ertugliflozin according to the therapeutic regimens of the included studies. **Scenario 1**: Ertugliflozin monotherapy (52 weeks) vs. placebo (26 weeks) + metformin alone (26 weeks): ertugliflozin showed higher costs ($364.00 > $97.73) and a lower rate of patients achieving target HbA1c levels (< 7%) (0.2696 < 0.2745), indicating ertugliflozin alone was dominated. **Scenario 2**: Ertugliflozin + metformin vs. glimepiride + metformin for 104 weeks. The former group was associated with higher costs ($1055.60 > $531.44) and lower effectiveness (0.2545 < 0.2828), indicating ertugliflozin plus metformin therapy was dominated compared with glimepiride + metformin. **Scenario 3**: Ertugliflozin + metformin vs. sitagliptin + metformin for 52 weeks: ertugliflozin group produced lower costs ($527.80 < $564.20) and lower effectiveness (0.2410 < 0.2672). The ICER value is $1390.04, which is lower than the WTP value. **Scenario 4**: Ertugliflozin + metformin vs. metformin monotherapy for 26 weeks: ertugliflozin add-on therapy increased the costs ($263.90 > $81.90) but earned better effectiveness (0.7452 > 0.1620). The ICER value was $312.07, which was lower than the WTP value. **Scenario 5**: Ertugliflozin + sitagliptin + metformin vs. sitagliptin + metformin (metformin: 1500 mg/d or 2000 mg/d) for 52 weeks: ertugliflozin add-on therapy augmented the costs (1500 mg/d metformin $928.20 > $564.20; 2000 mg/d metformin $982.8 > $618.8) and increased the rate of patients achieving target HbA1c levels (< 7%) (1500 mg/d metformin 0.4024 > 0.2672; 2000 mg/d metformin 0.5188 > 0.1373). Their ICER values were $2692.18 (1500 mg/d metformin) and $953.96 (2000 mg/d metformin), respectively, lower than the WTP value. Overall, variation of effects and cost did not reverse the cost-effectiveness of ertugliflozin through one-way sensitivity analyses. We failed to detect the influence of cost variation on the main results. However, ICERs were sensitive to the changes in effects. For instance, in scenario 1, when the effect of ertugliflozin attained the upper limit of 95% CI (0.3698), the ICER increased to $2795.30, indicating a dominant result than control.

**3. Discussion**

The global economic burden of type 2 diabetes in adults is enormous. Economic factors also matter when making clinical decisions. Therefore, the economic evaluation of hypoglycaemic agents is essential, especially for new drugs. A comprehensive economic evaluation using clinical trials and real-world evidence suggested that SGLT2 inhibitors (ertugliflozin were not included) were cost-saving and highly cost-effective because of the significantly reduced complication costs and raised quality-adjusted life years (McEwan et al., 2020). Individual SGLT2 inhibitors, such as dapagliflozin, canagliflozin, and empagliflozin, have also been economically assessed in China, incorporating the profile of randomised controlled trials. Recent reports showed that dapagliflozin might be a cost-effective treatment versus acarbose as monotherapy in Chinese patients with type 2 diabetes, but not metformin (Gu et al., 2016; Nian et al., 2020). Canagliflozin might be a cost-saving option relative to dapagliflozin for Chinese patients with type 2 diabetes who were inadequately controlled with metformin (Hou et al., 2019). Empagliflozin is a cost-effective alternative to glimepiride by applying a threshold of $30290 in China (Salem et al., 2021). To our knowledge, there has been no local economic assessment of ertugliflozin in China due to new market entry. The economic assessment of ertugliflozin contributed to determining whether ertugliflozin was a cost-effective alternative to other hypoglycaemic agents in Chinese patients with type 2 diabetes. The included RCTs did not report the economic outcomes of ertugliflozin; therefore, we performed a cost-effectiveness analysis versus other glucose-lowering drugs based on our meta-analysis. Generally, ertugliflozin appeared to be cost-effective in the two scenarios. One scenario is when ertugliflozin was added as dual therapy with metformin compared with metformin alone. Another was when ertugliflozin was added to sitagliptin and metformin. Even if the results need to be verified by more high-quality studies based on an economic model, combining therapy with ertugliflozin may contribute to the preferred economic effect.

**References**

Collinge, M., Ball, D.J., Bowman, C.J., Nilson, A.L., Radi, Z.A., and Vogel, W.M. (2018). Immunologic effects of chronic administration of tofacitinib, a Janus kinase inhibitor, in cynomolgus monkeys and rats - Comparison of juvenile and adult responses. *Regul Toxicol Pharmacol* 94**,** 306-322. doi: 10.1016/j.yrtph.2018.02.006.

Gu, S., Mu, Y., Zhai, S., Zeng, Y., Zhen, X., and Dong, H. (2016). Cost-Effectiveness of Dapagliflozin versus Acarbose as a Monotherapy in Type 2 Diabetes in China. *PLoS One* 11(11)**,** e0165629. doi: 10.1371/journal.pone.0165629.

Hou, X., Wan, X., and Wu, B. (2019). Cost-Effectiveness of Canagliflozin Versus Dapagliflozin Added to Metformin in Patients With Type 2 Diabetes in China. *Front Pharmacol* 10**,** 480. doi: 10.3389/fphar.2019.00480.

Kremer, J.M., Schiff, M., Muram, D., Zhong, J., Alam, J., and Genovese, M.C. (2018). Response to baricitinib therapy in patients with rheumatoid arthritis with inadequate response to csDMARDs as a function of baseline characteristics. *RMD Open* 4(1)**,** e000581. doi: 10.1136/rmdopen-2017-000581.

McEwan, P., Bennett, H., Khunti, K., Wilding, J., Edmonds, C., Thuresson, M., et al. (2020). Assessing the cost-effectiveness of sodium-glucose cotransporter-2 inhibitors in type 2 diabetes mellitus: A comprehensive economic evaluation using clinical trial and real-world evidence. *Diabetes Obes Metab* 22(12)**,** 2364-2374. doi: 10.1111/dom.14162.

Nian, H., Wan, X., Ma, J., Jie, F., and Wu, B. (2020). Economic evaluation of dapagliflozin versus metformin in Chinese patients whose diabetes is inadequately controlled with diet and exercise. *Cost Eff Resour Alloc* 18**,** 12. doi: 10.1186/s12962-020-00208-w.

Salem, A., Men, P., Ramos, M., Zhang, Y.J., Ustyugova, A., and Lamotte, M. (2021). Cost-effectiveness analysis of empagliflozin compared with glimepiride in patients with Type 2 diabetes in China. *J Comp Eff Res* 10(6)**,** 469-480. doi: 10.2217/cer-2020-0284.

Sathish, T., Oldenburg, B., Thankappan, K.R., Absetz, P., Shaw, J.E., Tapp, R.J., et al. (2020). Cost-effectiveness of a lifestyle intervention in high-risk individuals for diabetes in a low- and middle-income setting: Trial-based analysis of the Kerala Diabetes Prevention Program. *BMC Med* 18(1)**,** 251. doi: 10.1186/s12916-020-01704-9.

WHO (2001). World Health Organization Commission on Macroeconomics and Health. Macroeconomics and health: investing in health for economic development. Report of the Commission on Macroeconomics and Health. Geneva: World Health Organization.

**Supplementary tables**

**Table S1. Search strategy used on 31 July 2021**

| **Literature databases** | **Search items** | **Items found** |
| --- | --- | --- |
| Pubmed | (((randomized controlled trial) OR (controlled clinical trial)) OR (clinical trial)) AND ("ertugliflozin"[Title/Abstract] OR "steglatro"[Title/Abstract] OR "steglujan"[Title/Abstract] OR "segluromet"[Title/Abstract] OR "MK-8835"[Title/Abstract] OR "PF-04971729"[Title/Abstract] OR "PF-4971729"[Title/Abstract]) | 46 |
| Embase | (ertugliflozin:ti,ab,kw OR steglatro:ti,ab,kw OR steglujan:ti,ab,kw OR segluromet:ti,ab,kw OR 'mk-8835':ti,ab,kw OR 'pf-04971729':ti,ab,kw OR 'pf-4971729':ti,ab,kw) AND (randomized AND controlled AND trial OR (controlled AND clinical AND trial) OR (clinical AND trial)) AND [embase]/lim NOT ([embase]/lim AND [medline]/lim) | 85 |
| Cochrance | ((ertugliflozin):ti,ab,kw OR (steglatro):ti,ab,kw OR (steglujan):ti,ab,kw OR (segluromet):ti,ab,kw OR (MK-8835):ti,ab,kw OR (PF-04971729):ti,ab,kw OR (PF-4971729):ti,ab,kw) AND (randomized controlled trial OR (controlled clinical trial) OR (clinical trial)) | 105 |
| Overall |  | 236 |
| Duplicates |  | 45 |

**Table S2. Definition of adverse events (AEs) included in the analysis**

| **Definition** | **AEs related to study drug** | **Symptomatic hypoglycaemia** | **Genital mycotic infection** | **Urinary tract infection** | **Hypovolaemia** | **Any AEs/Serious AEs/Deaths/AEs leading to discontinuation** |
| --- | --- | --- | --- | --- | --- | --- |
| **Amin, 2015** | Adverse events were coded according to the Medical Dictionary for Regulatory Activities (MedDRA, version 13.1). | | | | | |
| **Aronson, 2018** | Determined by the investigator to be related to the study drug | Consisted of episodes with clinical symptoms of hypoglycaemia reported by the investigator | Based on pre-specified sponsor-generated customised MedDRA queries (CMQs) of preferred terms. | | | Undefined |
| **Dagogo, 2018** | As reported by the investigator | Defined as episodes with clinical symptoms reported by the investigator as hypoglycaemia | Undefined | | | |
| **Gallo, 2019** | Assessed as related to the study drug by the investigator | Identified according to pre-specified sponsor-generated customized MedDRA queries of preferred terms | | | | Undefined |
| **Grunberger, 2018** | Determined by the investigator to be related to the study drug | Event with clinical symptoms reported by the investigator as hypoglycaemia | Undefined | | | |
| **Hollander, 2019** | As reported by the investigator | Defined as episodes with clinical symptoms reported by the investigator | Undefined | | Defined on the basis of prespecified sponsor-generated Custom MedDRA Query (CMQ) of preferred terms associated with hypovolemia | Undefined |
| **Ji, 2019** | Assessed as related to the study drug by the investigator | Undefined | Undefined | | Undefined | Undefined |
| **Miller, 2018** | AEs reported by the investigator | Defined as episodes with clinical symptoms reported by the investigator as hypoglycaemia | Undefined | | Undefined | Undefined |
| **Pratley, 2018** | Determined by the investigator to be related to the study drug | Episodes with clinical symptoms reported by the investigator as hypoglycaemia | Selected from pre-specified sponsor-generated customized Medical Dictionary for Regulatory Activities queries of preferred terms | | | Undefined |

**Table S3. Leave-one-out sensitivity analysis for main efficacy outcomes of ertugliflozin**

| **Study omitted** | **WMD/RR** | **95%CI** |
| --- | --- | --- |
| **HbA1c%** | WMD | 95%CI |
| No omission | -0.452 | -0.774 to -0.129 |
| Amin, 2015 | -0.458 | -0.810 to -0.106 |
| Aronson, 2018 | -0.515 | -0.864 to -0.166 |
| Dagogo, 2018 | -0.408 | -0.753 to -0.063 |
| Gallo, 2019 | -0.493 | -0.847 to -0.139 |
| Grunberger, 2018 | -0.502 | -0.857 to -0.148 |
| Hollander, 2019 | -0.523 | -0.829 to -0.218 |
| Ji, 2019 | -0.413 | -0.763 to -0.064 |
| Miller, 2018 | -0.360 | -0.674 to -0.046 |
| Pratley, 2018 | -0.394 | -0.731 to -0.057 |
| **FPG** | WMD | 95%CI |
| No omission | -0.870 | -1.418 to -0.322 |
| Amin, 2015 | -0.902 | -1.488 to -0.317 |
| Aronson, 2018 | -0.980 | -1.586 to -0.373 |
| Dagogo, 2018 | -0.774 | -1.342 to -0.206 |
| Gallo, 2019 | -0.954 | -1.594 to -0.314 |
| Grunberger, 2018 | -0.993 | -1.575 to -0.412 |
| Hollander, 2019 | -0.959 | -1.592 to -0.327 |
| Ji, 2019 | -0.769 | -1.309 to -0.228 |
| Miller, 2018 | -0.680 | -1.202 to -0.159 |
| Pratley, 2018 | -0.816 | -1.396 to -0.236 |
| **Body weight** | WMD | 95%CI |
| No omission | -1.774 | -2.601 to -0.946 |
| Amin, 2015 | -1.774 | -2.601 to -0.946 |
| Aronson, 2018 | -2.038 | -3.052 to -1.025 |
| Dagogo, 2018 | -1.711 | -2.599 to -0.824 |
| Gallo, 2019 | -1.931 | -3.189 to -0.672 |
| Grunberger, 2018 | -1.784 | -2.677 to -0.892 |
| Hollander, 2019 | -1.392 | -2.012 to -0.772 |
| Ji, 2019 | -1.755 | -2.677 to -0.832 |
| Miller, 2018 | -1.734 | -2.625 to -0.843 |
| Pratley, 2018 | -1.810 | -2.679 to -0.941 |
| **SBP** | WMD | 95%CI |
| No omission | -2.572 | -3.573 to -1.571 |
| Amin, 2015 | -2.494 | -3.527 to -1.462 |
| Aronson, 2018 | -3.214 | -4.979 to -1.448 |
| Dagogo, 2018 | -2.274 | -3.257 to -1.291 |
| Gallo, 2019 | -3.220 | -5.063 to -1.378 |
| Grunberger, 2018 | -2.512 | -3.536 to -1.488 |
| Hollander, 2019 | -2.310 | -3.280 to -1.341 |
| Ji, 2019 | -2.251 | -3.223 to -1.279 |
| Miller, 2018 | -2.287 | -3.275 to -1.300 |
| Pratley, 2018 | -2.763 | -3.847 to -1.679 |
| **DBP** | WMD | 95%CI |
| No omission | -1.152 | -2.002 to -0.303 |
| Amin, 2015 | -0.850 | -1.555 to -0.145 |
| Dagogo, 2018 | -1.196 | -2.156 to -0.237 |
| Gallo, 2019 | -1.393 | -2.122 to -0.664 |
| Hollander, 2019 | -1.103 | -2.063 to -0.143 |
| Ji, 2019 | -1.116 | -2.061 to -0.171 |
| Miller, 2018 | -1.058 | -1.952 to -0.165 |
| Pratley, 2018 | -1.343 | -2.328 to -0.357 |
| **Patients of HbA1c% < 7%** | RR | 95%CI |
| No omission | 1.512 | 1.073 to 1.951 |
| Amin, 2015 | 1.446 | 1.007 to 1.885 |
| Aronson, 2018 | 1.787 | 1.207 to 2.366 |
| Dagogo, 2018 | 1.330 | 0.936 to 1.723 |
| Gallo, 2019 | 1.531 | 1.030 to 2.032 |
| Hollander, 2019 | 1.853 | 1.247 to 2.459 |
| Ji, 2019 | 1.325 | 0.945 to 1.705 |
| Miller, 2018 | 1.439 | 1.024 to 1.853 |
| Pratley, 2018 | 1.772 | 1.165 to 2.379 |

HbA1c, glycated haemoglobin; FPG, fasting plasma glucose; SBP, systolic blood pressure; DBP, diastolic blood pressure; WMD, weighted mean difference; RR, risk ratio.

**Table S4. Subgroup analysis of main efficacy outcomes of ertugliflozin versus control using a fixed-effect model**

| **Subgroup** | | **No. S** | **WMD** | **95%CI** | ***I*^2^** | **PI** |
| --- | --- | --- | --- | --- | --- | --- |
| **Different dosages** | | | | | | |
| HbA1c% | 5mg | 8 | -0.288 | -0.370 to -0.207 | 94.4% | 0.886 |
|  | 15mg | 8 | -0.341 | -0.421 to -0.260 | 93.2% |  |
| FPG | 5mg | 8 | -0.515 | -0.634 to -0.396 | 95.2% | 0.738 |
|  | 15mg | 8 | -0.618 | -0.739 to -0.497 | 93.7% |  |
| Body weight | 5mg | 8 | -0.978 | -1.124 to -0.832 | 96.0% | 0.931 |
|  | 15mg | 8 | -0.935 | -1.081 to -0.790 | 96.0% |  |
| SBP | 5mg | 8 | -0.267 | -0.424 to -0.110 | 88.0% | 0.516 |
|  | 15mg | 8 | 0.044 | -0.111 to 0.199 | 87.7% |  |
| DBP | 5mg | 6 | -0.230 | -0.418 to -0.042 | 61.6% | 0.579 |
|  | 15mg | 6 | -0.143 | -0.331 to 0.045 | 38.3% |  |
| **Different follow-ups** | | | | | | |
| HbA1c% | ≤26 weeks | 3 | -0.789 | -0.901 to -0.676 | 88.6% | 0.061 |
|  | >26 weeks | 6 | -0.175 | -0.247 to -0.104 | 95.6% |  |
| FPG | ≤26 weeks | 3 | -1.696 | -1.899 to -1.494 | 89.2% | 0.023 |
|  | >26 weeks | 6 | -0.362 | -0.470 to -0.254 | 94.3% |  |
| Body weight | ≤26 weeks | 2 | -1.929 | -2.243 to -1.615 | 0% | 0.677 |
|  | >26 weeks | 6 | -0.779 | -0.890 to -0.668 | 98.0% |  |
| SBP | ≤26 weeks | 3 | -3.961 | -4.952 to -2.791 | 50.6% | 0.006 |
|  | >26 weeks | 6 | -0.064 | -0.160 to 0.032 | 92.5% |  |
| DBP | ≤26 weeks | 3 | -2.062 | -2.741 to -1.383 | 18.7% | 0.006 |
|  | >26 weeks | 4 | -0.157 | -0.289 to -0.025 | 69.7% |  |
| **Different controls** | | | | | | |
| HbA1c% | placebo | 5 | -0.665 | -0.752 to 0.578 | 93.0% | 0.039 |
|  | active | 4 | -0.060 | -0.144 to 0.023 | 94.9% |  |
| FPG | placebo | 5 | -1.532 | -1.694 to -1.370 | 92.4% | 0.006 |
|  | active | 4 | -0.191 | -0.309 to -0.074 | 73.1% |  |
| Body weight | placebo | 4 | -1.954 | -2.221 to -1.686 | 0% | 0.565 |
|  | active | 4 | -0.717 | -0.831 to -0.603 | 98.7% |  |
| SBP | placebo | 5 | -4.110 | -4.967 to -3.253 | 19.3% | < 0.001 |
|  | active | 4 | -0.050 | -0.146 to 0.046 | 92.3% |  |
| DBP | placebo | 4 | -1.810 | -2.411 to -1.209 | 38.8% | 0.037 |
|  | active | 3 | -0.149 | -0.281 to -0.016 | 76.8% |  |
| **Subgroup** | | **No. S** | **RR** | **95%CI** | ***I^2^*** | **PI** |
| **Different dosages** | | | | | | |
| Patients achieving HbA1c <7% | 5mg | 7 | 1.069 | 0.910 to 1.228 | 64.9% | 0.641 |
|  | 15mg | 7 | 1.125 | 0.951 to 1.299 | 69.2% |  |
| **Different follow-ups** | | | | | | |
| Patients achieving HbA1c <7% | ≤26 weeks | 3 | 3.826 | 2.463 to 5.190 | 41.0% | 0.005 |
|  | >26 weeks | 5 | 1.053 | 0.918 to 1.188 | 76.6% |  |
| **Different controls** | | | | | | |
| Patients achieving HbA1c <7% | placebo | 4 | 3.808 | 2.742 to 4.873 | 11.6% | < 0.001 |
|  | active | 4 | 1.036 | 0.901 to 1.171 | 58.4% |  |

No. S, numbers of studies; WMD, weighted mean difference; PI, *P* for interaction; RR, risk ratio; HbA1c, glycated haemoglobin; FPG, fasting plasma glucose; SBP, systolic blood pressure; DBP, diastolic blood pressure.

**Table S5. Summarized adverse events of interest in included studies**

| **Adverse Events of interest** | **Ertugliflozin (n=3959)** | **Control (n=1669)** |
| --- | --- | --- |
| Any AEs | 2517 (63.58%) | 1097 (65.73%) |
| AEs related to study drug | 761(19.22%) | 286 (17.14%) |
| Serious AEs | 265 (6.69%) | 104 (6.23%) |
| Deaths | 22 (0.56%) | 7 (0.42%) |
| AEs leading to discontinuation | 171 (4.32%) | 65 (3.89%) |
| Genital mycotic infection | 261 (6.59%) | 24 (1.44%) |
| Urinary tract infection | 274 (6.92%) | 128 (7.67%) |
| Symptomatic hypoglycaemia | 212 (5.35%) | 181 (10.84%) |
| Hypovolaemia | 62 (1.66%) | 19 (1.18%) |

Data are *n* (%), with *n* presented the number of patients occurred the adverse events in that groups.

**Table S6. Subgroup analysis of main safety outcomes of ertugliflozin versus control using a fixed-effect model**

| **Subgroup** | | **No. S** | **RR** | **95%CI** | ***I^2^*** | **PI** |
| --- | --- | --- | --- | --- | --- | --- |
| **Different dosages** | | | | | | |
| Any AEs | 5mg | 8 | 0.992 | 0.945 to 1.041 | 0 | 0.586 |
|  | 15mg | 8 | 0.976 | 0.930 to 1.025 | 0 |  |
| AEs related to study drug | 5mg | 8 | 1.137 | 0.985 to 1.313 | 53.6% | 0.921 |
|  | 15mg | 8 | 1.172 | 1.015 to 1.353 | 59.9% |  |
| Serious AEs | 5mg | 8 | 1.238 | 0.970 to 1.580 | 0 | 0.489 |
|  | 15mg | 8 | 1.084 | 0.843 to 1.394 | 8.2% |  |
| Deaths | 5mg | 4 | 1.641 | 0.665 to 4.049 | 29.1 | 0.875 |
|  | 15mg | 4 | 1.266 | 0.489 to 3.278 | 0 |  |
| AEs leading to discontinuation | 5mg | 8 | 1.153 | 0.837 to 1.588 | 0 | 0.980 |
|  | 15mg | 8 | 1.139 | 0.824 to 1.573 | 0 |  |
| Genital mycotic infection | 5mg | 8 | 4.739 | 3.037 to 7.396 | 1.8% | 0.882 |
|  | 15mg | 8 | 5.079 | 3.260 to 7.914 | 26.2% |  |
| Urinary tract infection | 5mg | 8 | 0.885 | 0.696 to 1.126 | 39.5% | 0.490 |
|  | 15mg | 8 | 0.985 | 0.780 to 1.245 | 10.2% |  |
| Symptomatic hypoglycaemia | 5mg | 8 | 0.501 | 0.396 to 0.634 | 83.8% | 0.912 |
|  | 15mg | 8 | 0.554 | 0.440 to 0.697 | 75.1% |  |
| Hypovolaemia | 5mg | 8 | 1.550 | 0.881 to 2.728 | 19.1% | 0.934 |
|  | 15mg | 8 | 1.393 | 0.781 to 2.485 | 1.5% |  |
| **Different follow-ups** | | | | | | |
| Any AEs | ≤26 weeks | 3 | 0.973 | 0.859 to 1.102 | 0 | 0.768 |
|  | >26 weeks | 6 | 0.986 | 0.944 to 1.030 | 0 |  |
| AEs related to study drug | ≤26 weeks | 3 | 1.235 | 0.873 to 1.745 | 0 | 0.761 |
|  | >26 weeks | 6 | 1.139 | 0.996 to 1.302 | 64.3% |  |
| Serious AEs | ≤26 weeks | 3 | 1.703 | 0.763 to 3.797 | 65.4% | 0.845 |
|  | >26 weeks | 6 | 1.123 | 0.896 to 1.408 | 0 |  |
| Deaths | >26 weeks | 5 | 1.350 | 0.605 to 3.015 | 0 |  |
| AEs leading to discontinuation | ≤26 weeks | 3 | 0.771 | 0.302 to 1.964 | 0 | 0.314 |
|  | >26 weeks | 6 | 1.202 | 0.895 to 1.615 | 0 |  |
| Genital mycotic infection | ≤26 weeks | 3 | 1.983 | 0.760 to 5.178 | 0 | 0.112 |
|  | >26 weeks | 6 | 5.477 | 3.439 to 8.720 | 23.4 |  |
| Urinary tract infection | ≤26 weeks | 3 | 0.828 | 0.405 to 1.692 | 0 | 0.810 |
|  | >26 weeks | 6 | 0.932 | 0.754 to 1.154 | 27.8% |  |
| Symptomatic hypoglycaemia | ≤26 weeks | 3 | 4.066 | 1.116 to 14.813 | 0 | 0.329 |
|  | >26 weeks | 6 | 0.479 | 0.394 to 0.582 | 87.2% |  |
| Hypovolaemia | ≤26 weeks | 2 | 1.244 | 0.244 to 6.351 | 0 | 0.898 |
|  | >26 weeks | 6 | 1.499 | 0.868 to 2.588 | 51.9% |  |
| **Different controls** | | | | | | |
| Any AEs | placebo | 5 | 0.964 | 0.897 to 1.037 | 0 | 0.533 |
|  | active | 4 | 0.995 | 0.946 to 1.046 | 0 |  |
| AEs related to study drug | placebo | 5 | 1.256 | 0.988 to 1.596 | 0 | 0.571 |
|  | active | 4 | 1.112 | 0.961 to 1.287 | 76.6% |  |
| Serious AEs | placebo | 5 | 1.222 | 0.863 to 1.732 | 30.8% | 0.930 |
|  | active | 4 | 1.130 | 0.855 to 1.493 | 0 |  |
| Deaths | active | 4 | 1.470 | 0.538 to 4.019 | 0 |  |
| AEs leading to discontinuation | placebo | 5 | 1.006 | 0.604 to 1.677 | 0 | 0.549 |
|  | active | 4 | 1.225 | 0.875 to 1.717 | 2.8% |  |
| Genital mycotic infection | placebo | 5 | 3.428 | 1.653 to 7.108 | 0 | 0.272 |
|  | active | 4 | 5.394 | 3.249 to 8.955 | 35.6% |  |
| Urinary tract infection | placebo | 5 | 0.717 | 0.496 to 1.038 | 0 | 0.156 |
|  | active | 4 | 1.026 | 0.802 to 1.311 | 27.9% |  |
| Symptomatic hypoglycaemia | placebo | 5 | 1.112 | 0.802 to 1.540 | 19.9 | 0.075 |
|  | active | 4 | 0.348 | 0.271 to 0.445 | 82.7% |  |
| Hypovolaemia | placebo | 4 | 1.681 | 0.623 to 4.537 | 33.4 | 0.917 |
|  | active | 4 | 1.399 | 0.762 to 2.568 | 53.8% |  |

No. S, numbers of studies; RR, risk ratio; PI, *P* for interaction; AEs, adverse events.

**Table S7. Quality assessment results of included randomized controlled trials**

| **Study** | **Random sequence generation** | **Allocation concealment** | **Blinding of participants and personnel** | **Blinding of outcome assessment** | **Incomplete outcome data** | **Selective reporting** |
| --- | --- | --- | --- | --- | --- | --- |
| Amin, 2015 | L | U | L | U | L | L |
| Aronson, 2018 | L | L | L | L | L | L |
| Dagogo, 2018 | L | U | L | L | L | L |
| Gallo, 2019 | L | L | L | L | L | L |
| Grunberger, 2018 | L | L | L | L | L | L |
| Hollander, 2019 | L | L | L | L | L | L |
| Ji, 2019 | L | L | L | L | L | L |
| Miller, 2018 | L | L | L | L | L | L |
| Pratley, 2018 | L | L | L | L | L | L |

L: low risk; U: unclear risk.

**Table S8. PRISMA checklists**

| **Section and Topic** | **Item #** | **Checklist item** | **Location where item is reported** |
| --- | --- | --- | --- |
| **TITLE** | | |  |
| Title | 1 | Identify the report as a systematic review. | P1 |
| **ABSTRACT** | | |  |
| Abstract | 2 | See the PRISMA 2020 for Abstracts checklist. | P2 |
| **INTRODUCTION** | | |  |
| Rationale | 3 | Describe the rationale for the review in the context of existing knowledge. | P2 |
| Objectives | 4 | Provide an explicit statement of the objective(s) or question(s) the review addresses. | P2-3 |
| **METHODS** | | |  |
| Eligibility criteria | 5 | Specify the inclusion and exclusion criteria for the review and how studies were grouped for the syntheses. | P3-4 |
| Information sources | 6 | Specify all databases, registers, websites, organisations, reference lists and other sources searched or consulted to identify studies. Specify the date when each source was last searched or consulted. | P3-4 |
| Search strategy | 7 | Present the full search strategies for all databases, registers and websites, including any filters and limits used. | P3-4 |
| Selection process | 8 | Specify the methods used to decide whether a study met the inclusion criteria of the review, including how many reviewers screened each record and each report retrieved, whether they worked independently, and if applicable, details of automation tools used in the process. | P3-4 |
| Data collection process | 9 | Specify the methods used to collect data from reports, including how many reviewers collected data from each report, whether they worked independently, any processes for obtaining or confirming data from study investigators, and if applicable, details of automation tools used in the process. | P3-4 |
| Data items | 10a | List and define all outcomes for which data were sought. Specify whether all results that were compatible with each outcome domain in each study were sought (e.g. for all measures, time points, analyses), and if not, the methods used to decide which results to collect. | P3-4 |
|  | 10b | List and define all other variables for which data were sought (e.g. participant and intervention characteristics, funding sources). Describe any assumptions made about any missing or unclear information. | P3-4 |
| Study risk of bias assessment | 11 | Specify the methods used to assess risk of bias in the included studies, including details of the tool(s) used, how many reviewers assessed each study and whether they worked independently, and if applicable, details of automation tools used in the process. | P4 |
| Effect measures | 12 | Specify for each outcome the effect measure(s) (e.g. risk ratio, mean difference) used in the synthesis or presentation of results. | P4 |
| Synthesis methods | 13a | Describe the processes used to decide which studies were eligible for each synthesis (e.g. tabulating the study intervention characteristics and comparing against the planned groups for each synthesis (item #5)). | P3-4 |
|  | 13b | Describe any methods required to prepare the data for presentation or synthesis, such as handling of missing summary statistics, or data conversions. | P3-4 |
|  | 13c | Describe any methods used to tabulate or visually display results of individual studies and syntheses. | P3-4 |
|  | 13d | Describe any methods used to synthesize results and provide a rationale for the choice(s). If meta-analysis was performed, describe the model(s), method(s) to identify the presence and extent of statistical heterogeneity, and software package(s) used. | P3-4 |
|  | 13e | Describe any methods used to explore possible causes of heterogeneity among study results (e.g. subgroup analysis, meta-regression). | P3-4 |
|  | 13f | Describe any sensitivity analyses conducted to assess robustness of the synthesized results. | P3-4 |
| Reporting bias assessment | 14 | Describe any methods used to assess risk of bias due to missing results in a synthesis (arising from reporting biases). | P3-4 |
| Certainty assessment | 15 | Describe any methods used to assess certainty (or confidence) in the body of evidence for an outcome. | P3-4 |
| **RESULTS** | | |  |
| Study selection | 16a | Describe the results of the search and selection process, from the number of records identified in the search to the number of studies included in the review, ideally using a flow diagram. | P4-5 |
|  | 16b | Cite studies that might appear to meet the inclusion criteria, but which were excluded, and explain why they were excluded. | P4-5 |
| Study characteristics | 17 | Cite each included study and present its characteristics. | P4-5 |
| Risk of bias in studies | 18 | Present assessments of risk of bias for each included study. | P6 |
| Results of individual studies | 19 | For all outcomes, present, for each study: (a) summary statistics for each group (where appropriate) and (b) an effect estimate and its precision (e.g. confidence/credible interval), ideally using structured tables or plots. | P5-6 |
| Results of syntheses | 20a | For each synthesis, briefly summarise the characteristics and risk of bias among contributing studies. | P5-6 |
|  | 20b | Present results of all statistical syntheses conducted. If meta-analysis was done, present for each the summary estimate and its precision (e.g. confidence/credible interval) and measures of statistical heterogeneity. If comparing groups, describe the direction of the effect. | P5-6 |
|  | 20c | Present results of all investigations of possible causes of heterogeneity among study results. | P5-6 |
|  | 20d | Present results of all sensitivity analyses conducted to assess the robustness of the synthesized results. | P5-6 |
| Reporting biases | 21 | Present assessments of risk of bias due to missing results (arising from reporting biases) for each synthesis assessed. | P6 |
| Certainty of evidence | 22 | Present assessments of certainty (or confidence) in the body of evidence for each outcome assessed. | P5-6 |
| **DISCUSSION** | | |  |
| Discussion | 23a | Provide a general interpretation of the results in the context of other evidence. | P7-9 |
|  | 23b | Discuss any limitations of the evidence included in the review. | P9 |
|  | 23c | Discuss any limitations of the review processes used. | P9 |
|  | 23d | Discuss implications of the results for practice, policy, and future research. | P9 |
| **OTHER INFORMATION** | | |  |
| Registration and protocol | 24a | Provide registration information for the review, including register name and registration number, or state that the review was not registered. | P3 |
|  | 24b | Indicate where the review protocol can be accessed, or state that a protocol was not prepared. |  |
|  | 24c | Describe and explain any amendments to information provided at registration or in the protocol. |  |
| Support | 25 | Describe sources of financial or non-financial support for the review, and the role of the funders or sponsors in the review. | P9 |
| Competing interests | 26 | Declare any competing interests of review authors. | P9 |
| Availability of data, code and other materials | 27 | Report which of the following are publicly available and where they can be found: template data collection forms; data extracted from included studies; data used for all analyses; analytic code; any other materials used in the review. | P9 |

*From:*  Page MJ, McKenzie JE, Bossuyt PM, Boutron I, Hoffmann TC, Mulrow CD, et al. The PRISMA 2020 statement: an updated guideline for reporting systematic reviews. BMJ 2021;372:n71. doi: 10.1136/bmj.n71

For more information, visit: <http://www.prisma-statement.org/>

**Table S9. The costs data of medicines per day**

| **Medicines** | **Price (RMB/dosage)** | **Price (USD/dosage)** | **Dosage range (mg/day)** | **Cost range (USD/day)** |
| --- | --- | --- | --- | --- |
| Ertugliflozin | 3.46/5mg | 0.50/5mg | 5-15 | 0.50-1.50 |
| Metformin | 1.05/500mg | 0.15/500mg | 1500-2000 | 0.45-0.60 |
| Sitagliptin | 7.57/100mg | 1.10/100mg | 100 | 1.10 |
| Glimepiride | 1.12/2mg | 0.16/2mg | 3.5 | 0.28 |

1 USD = 6.8974 RMB

**Table S10. Results of cost-effectiveness and sensitivity analyses (the percentage of qualified HbA1c as effective index)**

| **Cost-effectiveness analysis** | **The percentage of qualified HbA1c** | | **Cost** | | **ICER(ΔC/ΔE)** |
| --- | --- | --- | --- | --- | --- |
|  | Intervention | Control | Intervention | Control |  |
| **Mono [Aronson, 2018] 52 weeks ^a^** | | | | | |
| Baseline value | 0.2696 | 0.2745 | 364.00 | 97.73 | Dominated ^#^ |
| Upper limit of 95% CI | 0.3698 | 0.2745 | 364.00 | 97.73 | 2795.30 |
| Lower limit of 95% CI | 0.1963 | 0.2745 | 364.00 | 97.73 | Dominated ^#^ |
| Increase 10% cost | 0.2696 | 0.2745 | 400.40 | 97.73 | Dominated ^#^ |
| Increase 20% cost | 0.2696 | 0.2745 | 436.80 | 97.73 | Dominated ^#^ |
| Decrease 10% cost | 0.2696 | 0.2745 | 327.60 | 97.73 | Dominated ^#^ |
| Decrease 20% cost | 0.2696 | 0.2745 | 291.20 | 97.73 | Dominated ^#^ |
| **ERT + MET VS MET [Ji, 2019] 26 weeks** | | | | | |
| Baseline value | 0.7452 | 0.1620 | 263.90 | 81.90 | 312.07 |
| Upper limit of 95% CI | 1.0838 | 0.1620 | 263.90 | 81.90 | 197.44 |
| Lower limit of 95% CI | 0.4066 | 0.1620 | 263.90 | 81.90 | 744.01 |
| Increase 10% cost | 0.7452 | 0.1620 | 290.29 | 81.90 | 357.32 |
| Increase 20% cost | 0.7452 | 0.1620 | 316.68 | 81.90 | 402.57 |
| Decrease 10% cost | 0.7452 | 0.1620 | 237.51 | 81.90 | 266.82 |
| Decrease 20% cost | 0.7452 | 0.1620 | 211.12 | 81.90 | 221.57 |
| **ERT + MET VS SIT + MET [Pratley, 2018] 52 weeks ^b^** | | | | | |
| Baseline value | 0.2410 | 0.2672 | 527.80 | 564.20 | 1390.04 |
| Upper limit of 95% CI | 0.1860 | 0.2672 | 527.80 | 564.20 | 448.11 |
| Lower limit of 95% CI | 0.3121 | 0.2672 | 527.80 | 564.20 | Dominant ^*^ |
| Increase 10% cost | 0.2410 | 0.2672 | 580.58 | 564.20 | Dominated ^#^ |
| Increase 20% cost | 0.2410 | 0.2672 | 633.36 | 564.20 | Dominated ^#^ |
| Decrease 10% cost | 0.2410 | 0.2672 | 475.02 | 564.20 | 3405.61 |
| Decrease 20% cost | 0.2410 | 0.2672 | 422.24 | 564.20 | 5421.17 |
| **ERT + MET VS GLI + MET [Hollander, 2019] 104 weeks** | | | | | |
| Baseline value | 0.2545 | 0.2828 | 1055.60 | 531.44 | Dominated ^#^ |
| Upper limit of 95% CI | 0.3088 | 0.2828 | 1055.60 | 531.44 | 20146.36 |
| Lower limit of 95% CI | 0.2002 | 0.2828 | 1055.60 | 531.44 | Dominated ^#^ |
| Increase 10% cost | 0.2545 | 0.2828 | 1161.16 | 531.44 | Dominated ^#^ |
| Increase 20% cost | 0.2545 | 0.2828 | 1266.72 | 531.44 | Dominated ^#^ |
| Decrease 10% cost | 0.2545 | 0.2828 | 950.04 | 531.44 | Dominated ^#^ |
| Decrease 20% cost | 0.2545 | 0.2828 | 844.48 | 531.44 | Dominated ^#^ |
| **ERT + SIT + MET VS SIT + MET [Pratley, 2018] 52 weeks ^b, c^** | | | | | |
| Baseline value | 0.4024 | 0.2672 | 928.20 | 564.20 | 2692.18 |
| Upper limit of 95% CI | 0.3188 | 0.2672 | 928.20 | 564.20 | 7058.25 |
| Lower limit of 95% CI | 0.5082 | 0.2672 | 928.20 | 564.20 | 1510.25 |
| Increase 10% cost | 0.4024 | 0.2672 | 1021.02 | 564.20 | 3378.68 |
| Increase 20% cost | 0.4024 | 0.2672 | 1113.84 | 564.20 | 4065.19 |
| Decrease 10% cost | 0.4024 | 0.2672 | 835.38 | 564.20 | 2005.67 |
| Decrease 20% cost | 0.4024 | 0.2672 | 742.56 | 564.20 | 1319.17 |
| **ERT + SIT + MET VS SIT + MET [Dagogo, 2018] 52 weeks ^d^** | | | | | |
| Baseline value | 0.5188 | 0.1373 | 982.80 | 618.80 | 953.96 |
| Upper limit of 95% CI | 0.7535 | 0.1373 | 982.80 | 618.80 | 590.65 |
| Lower limit of 95% CI | 0.2841 | 0.1373 | 982.80 | 618.80 | 2478.50 |
| Increase 10% cost | 0.5188 | 0.1373 | 1081.08 | 618.80 | 1211.53 |
| Increase 20% cost | 0.5188 | 0.1373 | 1179.36 | 618.80 | 1469.09 |
| Decrease 10% cost | 0.5188 | 0.1373 | 884.52 | 618.80 | 696.39 |
| Decrease 20% cost | 0.5188 | 0.1373 | 786.24 | 618.80 | 438.82 |

HbA1c, glycated haemoglobin; Intervention, Intervention group; Control, Control group; ICER, incremental cost-effectiveness ratio; ERT, ertugliflozin; MET, metformin; SIT, sitagliptin; GLI, glimepiride; a, metformin was performed for 26 weeks in control group. b, results were analyzed separately by either combining with metformin or combining with sitagliptin and metformin, control group was sitagliptin and metformin. c, metformin: 1500 mg/d; d, metformin, 2000 mg/d. # Intervention was more costly and less effective than the control group. * Intervention was less costly and more effective than the control group.

**Table S11. Results of cost-effectiveness and sensitivity analyses (the decreased value of HbA1c % as effective index)**

| **Cost-effectiveness analysis** | **The decrease value of HbA1c %** | | **Cost** | | **ICER(ΔC/ΔE)** |
| --- | --- | --- | --- | --- | --- |
|  | Intervention | Control | Intervention | Control |  |
| **Mono [Aronson, 2018] 52 weeks ^a^** | | | | | |
| Baseline value | 0.9450 | 1.0000 | 364.00 | 97.73 | Dominated ^#^ |
| Upper limit of 95% CI | 2.2560 | 1.0000 | 364.00 | 97.73 | 212.00 |
| Lower limit of 95% CI | -0.3660 | 1.0000 | 364.00 | 97.73 | Dominated ^#^ |
| Increase 10% cost | 0.9450 | 1.0000 | 400.40 | 97.73 | Dominated ^#^ |
| Increase 20% cost | 0.9450 | 1.0000 | 436.80 | 97.73 | Dominated ^#^ |
| Decrease 10% cost | 0.9450 | 1.0000 | 327.60 | 97.73 | Dominated ^#^ |
| Decrease 20% cost | 0.9450 | 1.0000 | 291.20 | 97.73 | Dominated ^#^ |
| **ERT + MET VS MET [Ji, 2019] 26 weeks** | | | | | |
| Baseline value | 0.9500 | 0.2000 | 263.90 | 81.90 | 242.67 |
| Upper limit of 95% CI | 1.0480 | 0.2000 | 263.90 | 81.90 | 214.62 |
| Lower limit of 95% CI | 0.8520 | 0.2000 | 263.90 | 81.90 | 279.14 |
| Increase 10% cost | 0.9500 | 0.2000 | 290.29 | 81.90 | 277.85 |
| Increase 20% cost | 0.9500 | 0.2000 | 316.68 | 81.90 | 313.04 |
| Decrease 10% cost | 0.9500 | 0.2000 | 237.51 | 81.90 | 207.48 |
| Decrease 20% cost | 0.9500 | 0.2000 | 211.12 | 81.90 | 172.29 |
| **ERT + MET VS SIT + MET [Pratley, 2018] 52 weeks ^b^** | | | | | |
| Baseline value | 0.9500 | 0.8000 | 527.80 | 564.20 | Dominant ^*^ |
| Upper limit of 95% CI | 1.0560 | 0.8000 | 527.80 | 564.20 | Dominant ^*^ |
| Lower limit of 95% CI | 0.8440 | 0.8000 | 527.80 | 564.20 | Dominant ^*^ |
| Increase 10% cost | 0.9500 | 0.8000 | 580.58 | 564.20 | 109.20 |
| Increase 20% cost | 0.9500 | 0.8000 | 633.36 | 564.20 | 461.07 |
| Decrease 10% cost | 0.9500 | 0.8000 | 475.02 | 564.20 | Dominant ^*^ |
| Decrease 20% cost | 0.9500 | 0.8000 | 422.24 | 564.20 | Dominant ^*^ |
| **ERT + MET VS GLI + MET [Hollander, 2019] 104 weeks** | | | | | |
| Baseline value | 0.3500 | 0.4000 | 1055.60 | 531.44 | Dominated ^#^ |
| Upper limit of 95% CI | 0.4480 | 0.4000 | 1055.60 | 531.44 | 10920.00 |
| Lower limit of 95% CI | 0.2520 | 0.4000 | 1055.60 | 531.44 | Dominated ^#^ |
| Increase 10% cost | 0.3500 | 0.4000 | 1161.16 | 531.44 | Dominated ^#^ |
| Increase 20% cost | 0.3500 | 0.4000 | 1266.72 | 531.44 | Dominated ^#^ |
| Decrease 10% cost | 0.3500 | 0.4000 | 950.04 | 531.44 | Dominated ^#^ |
| Decrease 20% cost | 0.3500 | 0.4000 | 844.48 | 531.44 | Dominated ^#^ |
| **ERT + SIT + MET VS SIT + MET [Pratley, 2018] 52 weeks ^b, c^** | | | | | |
| Baseline value | 1.4000 | 0.8000 | 928.20 | 564.20 | 606.67 |
| Upper limit of 95% CI | 1.4830 | 0.8000 | 928.20 | 564.20 | 532.94 |
| Lower limit of 95% CI | 1.3170 | 0.8000 | 928.20 | 564.20 | 704.06 |
| Increase 10% cost | 1.4000 | 0.8000 | 1021.02 | 564.20 | 761.37 |
| Increase 20% cost | 1.4000 | 0.8000 | 1113.84 | 564.20 | 916.07 |
| Decrease 10% cost | 1.4000 | 0.8000 | 835.38 | 564.20 | 451.97 |
| Decrease 20% cost | 1.4000 | 0.8000 | 742.56 | 564.20 | 297.27 |
| **ERT + SIT + MET VS SIT + MET [Dagogo, 2018] 52 weeks ^d^** | | | | | |
| Baseline value | 0.8000 | 0.0000 | 982.80 | 618.80 | 455.00 |
| Upper limit of 95% CI | 0.9770 | 0.0000 | 982.80 | 618.80 | 372.57 |
| Lower limit of 95% CI | 0.6230 | 0.0000 | 982.80 | 618.80 | 584.27 |
| Increase 10% cost | 0.8000 | 0.0000 | 1081.08 | 618.80 | 577.85 |
| Increase 20% cost | 0.8000 | 0.0000 | 1179.36 | 618.80 | 700.70 |
| Decrease 10% cost | 0.8000 | 0.0000 | 884.52 | 618.80 | 332.15 |
| Decrease 20% cost | 0.8000 | 0.0000 | 786.24 | 618.80 | 209.30 |

HbA1c, glycated haemoglobin; Intervention, Intervention group; Control, Control group; ICER, incremental cost-effectiveness ratio; ERT, ertugliflozin; MET, metformin; SIT, sitagliptin; GLI, glimepiride; a, metformin was performed for 26 weeks in control group. b, results were analyzed separately by either combining with metformin or combining with sitagliptin and metformin, control group was sitagliptin and metformin. c, metformin: 1500mg/d; d, metformin, 2000mg/d. # Intervention was more costly and less effective than the control group. * Intervention was less costly and more effective than the control group.

**Supplementary Figures**

**Figure S1. Forest plots of ertugliflozin on efficacy outcomes (A: HbA1c%, B: FPG, and C: body weight)**


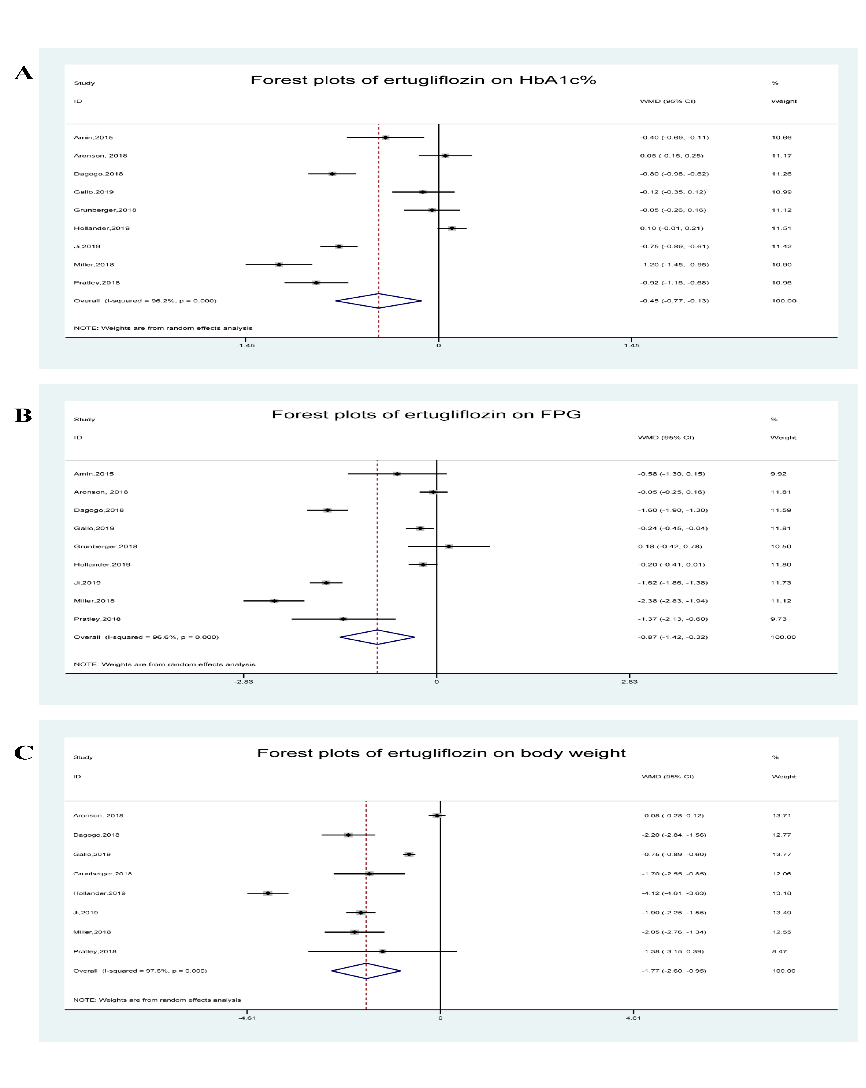


**Figure S2. Forest plots of ertugliflozin on efficacy outcomes (A: SBP, B: DBP, and C: proportion of patients achieving HbA1c <7%)**


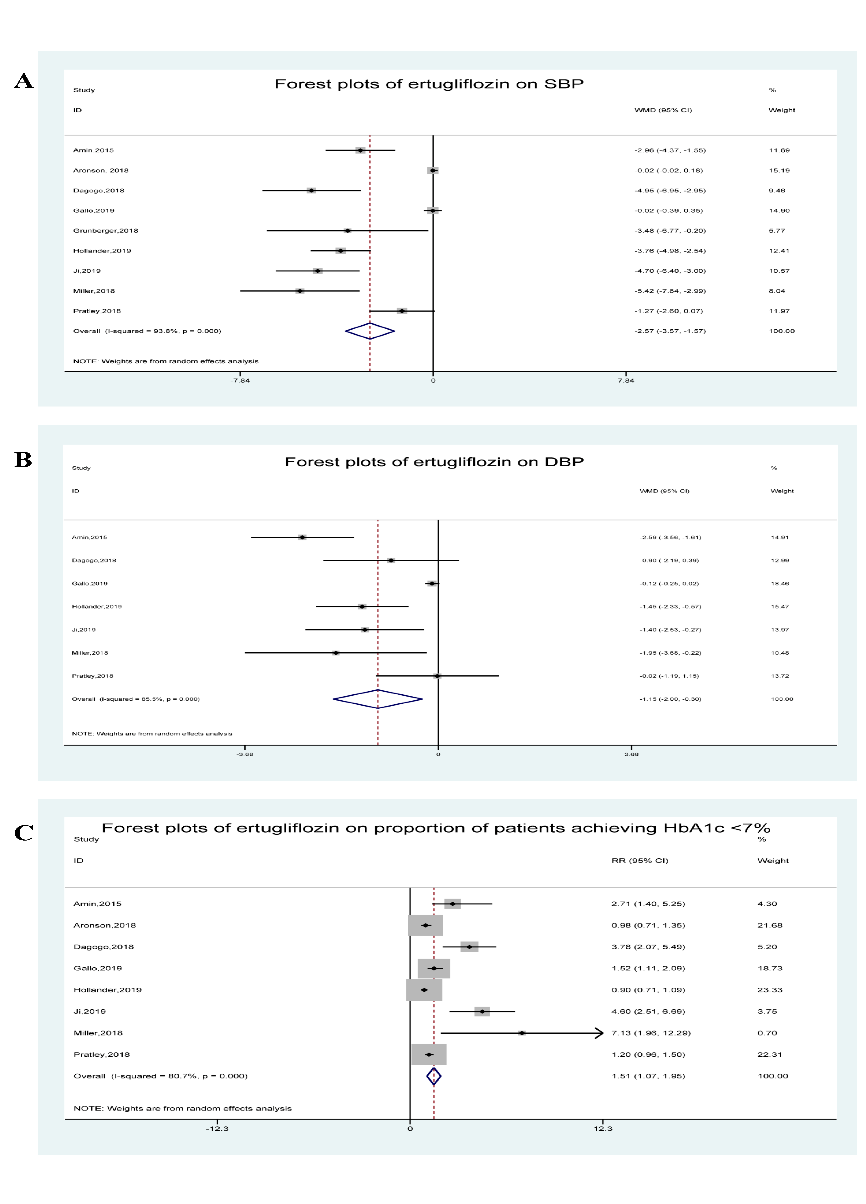


**Figure S3. Forest plots of ertugliflozin on safety outcomes (A: Any AEs, B: AEs related to study drug, and C: serious AEs)**


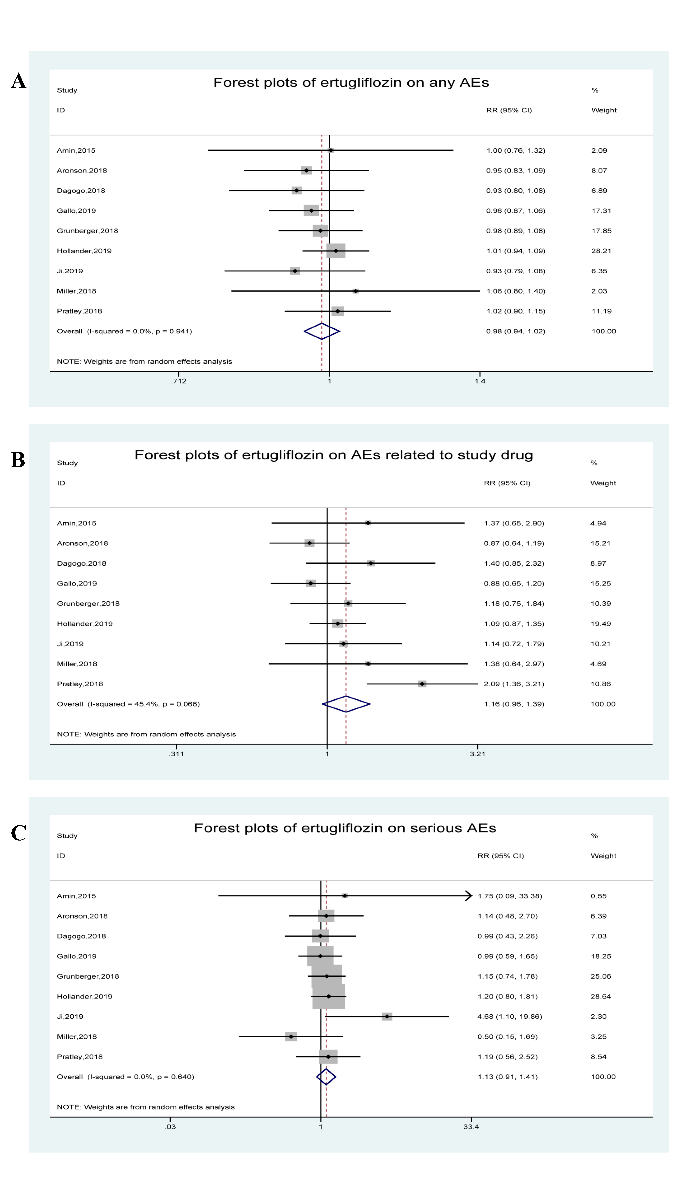


**Figure S4. Forest plots of ertugliflozin on safety outcomes (A: deaths, B: AEs leading to discontinuation, and C: GMI)**


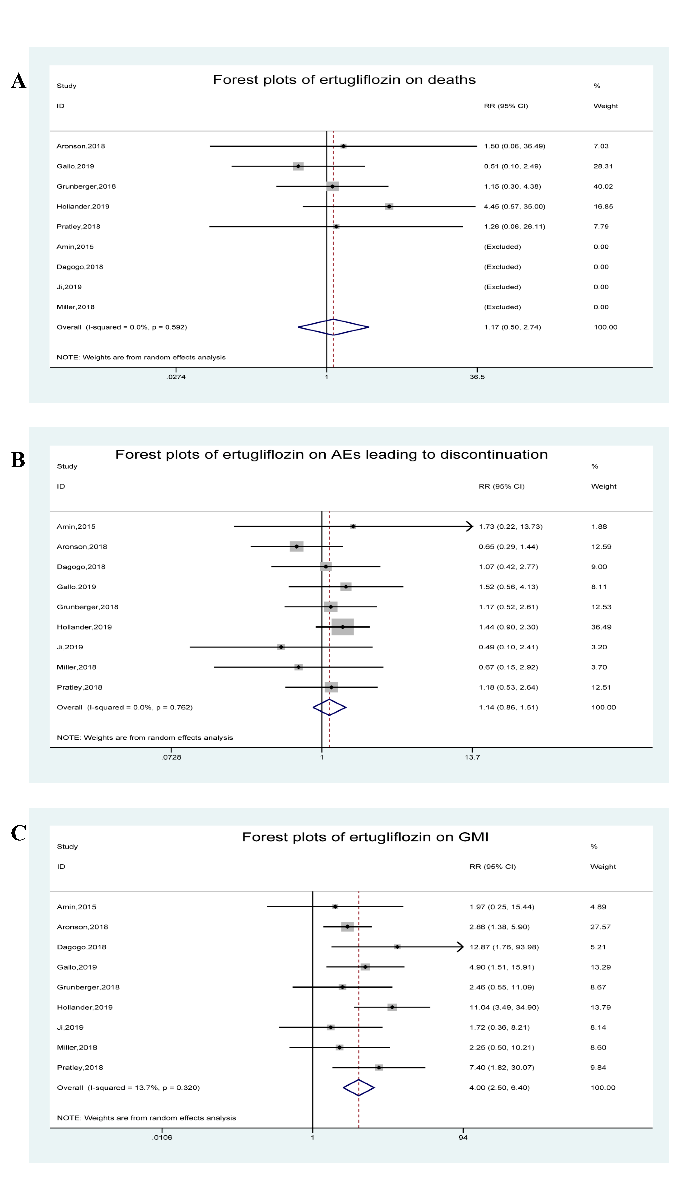


**Figure S5. Forest plots of ertugliflozin on safety outcomes (A: UTI, B: symptomatic hypoglycaemia, and C: hypovolaemia)**


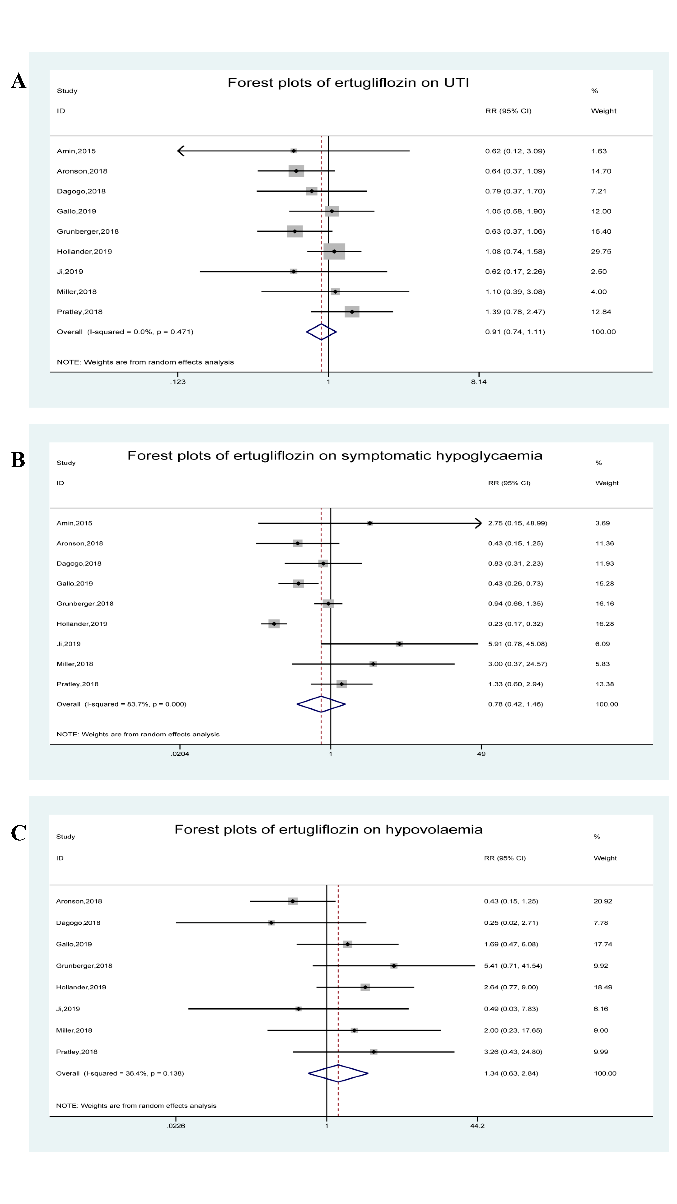


**Figure S6. Forest plots of ertugliflozin on efficacy and safety outcomes using a fixed-effect model**


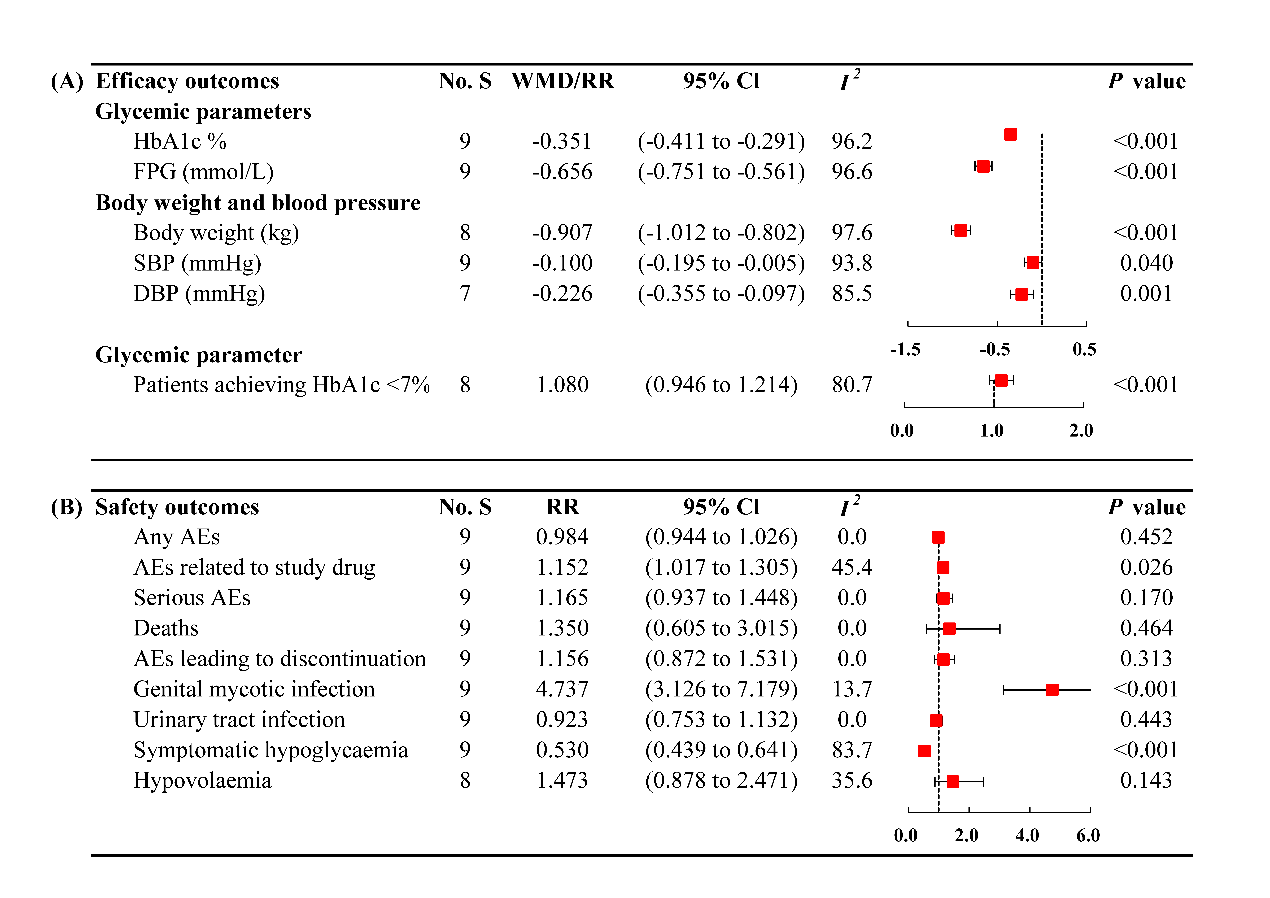


**Figure legend**

**Figure S6 Forest plots of ertugliflozin on efficacy and safety outcomes using a fixed-effect model**

No. S, numbers of studies; WMD, weighted mean difference; CI, confidence interval; *I^2^*, heterogeneity; HbA1c, glycated haemoglobin; FPG, fasting plasma glucose; SBP, systolic blood pressure; DBP, diastolic blood pressure; RR, risk ratio; AEs, adverse events.
